# Exergoeconomic Analysis and Optimization of a Biomass Integrated Gasification Combined Cycle Based on Externally Fired Gas Turbine, Steam Rankine Cycle, Organic Rankine Cycle, and Absorption Refrigeration Cycle

**DOI:** 10.3390/e26060511

**Published:** 2024-06-12

**Authors:** Jie Ren, Chen Xu, Zuoqin Qian, Weilong Huang, Baolin Wang

**Affiliations:** School of Naval Architecture, Ocean and Energy Power Engineering, Wuhan University of Technology, Wuhan 430063, China; j.ren@whut.edu.cn (J.R.); qzq@whut.edu.cn (Z.Q.); hwl220@whut.edu.cn (W.H.); wangbaolin1006@163.com (B.W.)

**Keywords:** biomass gasification, combined cooling and power, exergoeconomic analysis, externally fired gas turbine, absorption refrigeration cycle, multi-objective optimization

## Abstract

Adopting biomass energy as an alternative to fossil fuels for electricity production presents a viable strategy to address the prevailing energy deficits and environmental concerns, although it faces challenges related to suboptimal energy efficiency levels. This study introduces a novel combined cooling and power (CCP) system, incorporating an externally fired gas turbine (EFGT), steam Rankine cycle (SRC), absorption refrigeration cycle (ARC), and organic Rankine cycle (ORC), aimed at boosting the efficiency of biomass integrated gasification combined cycle systems. Through the development of mathematical models, this research evaluates the system’s performance from both thermodynamic and exergoeconomic perspectives. Results show that the system could achieve the thermal efficiency, exergy efficiency, and levelized cost of exergy (LCOE) of 70.67%, 39.13%, and 11.67 USD/GJ, respectively. The analysis identifies the combustion chamber of the EFGT as the component with the highest rate of exergy destruction. Further analysis on parameters indicates that improvements in thermodynamic performance are achievable with increased air compressor pressure ratio and gas turbine inlet temperature, or reduced pinch point temperature difference, while the LCOE can be minimized through adjustments in these parameters. Optimized operation conditions demonstrate a potential 5.7% reduction in LCOE at the expense of a 2.5% decrease in exergy efficiency when compared to the baseline scenario.

## 1. Introduction

Over recent decades, the swift expansion of industrial and economic activities has significantly increased the consumption of fossil fuels, leading to a series of global challenges including environmental degradation, energy scarcity, and the exhaustion of natural resources. Projections suggest that by 2035, the demand for global energy will surge by 37% from its 2013 levels [1]. Fossil fuels, which account for approximately 80% of global energy consumption, are major contributors to the emission of greenhouse gases (GHG) [2]. In response, the global community has enacted various environmental treaties, such as the Montreal Protocol, the Kyoto Protocol, and the Paris Agreement, aiming to reduce GHG emissions and the carbon footprint of nations. Against this backdrop, there is a growing emphasis on the exploration and adoption of renewable energy sources by governments worldwide.

Renewable energy solutions present viable alternatives for mitigating global warming, reducing carbon dioxide emissions, and enhancing the energy independence of countries that rely heavily on imported fossil fuels. Among these renewable resources, biomass stands out for its versatility and sustainability. Biomass derives from a variety of sources including forests, crops, agricultural by-products, and organic waste from industrial, human, and animal activities [3]. It undergoes conversion into more valuable products, including liquid and gaseous fuels, through thermochemical and biochemical processes. Specifically, biomass gasification, a process of thermochemical conversion through partial oxidation, emerges as an optimal strategy for converting biomass into a syngas composed of carbon monoxide, hydrogen, carbon dioxide, gaseous hydrocarbons, and water vapor, along with minor amounts of char and condensable compounds [4]. The biomass integrated gasification combined cycle (BIGCC) has been recognized for its environmental friendliness, operational efficiency, and economic viability in electricity generation, positioning it as a pivotal technology in the shift toward renewable energy sources [5,6].

The adoption of gas turbine (GT) cycles for power generation from bioenergy is on the rise. Nonetheless, employing biogas in traditional internally fired GT cycles presents unique challenges. Gas turbines, being precision machinery, demand the expensive gas cleanup systems for highly purified gas to avoid fuel injector blockage and turbine blade damage [7]. Furthermore, the syngas from biomass gasification, characterized by its low calorific value, necessitates a substantial air intake for combustion to reach desired turbine inlet temperatures that require major modifications of commercially available gas turbines to prevent compressor surge conditions. The above-mentioned problems could be conveniently solved by employing an externally fired gas turbine (EFGT) cycle [8]. The EFGT configuration, where combustion occurs externally at low pressure, enables the use of lower-grade biofuels. In this setup, the turbine is powered by hot compressed air which is heated to the requisite turbine inlet temperature by flue gases from an external combustor via a high-temperature heat exchanger (HTHE) [9]. This configuration, using clean air as the working fluid, not only mitigates maintenance demands but also prolongs the service life of the turbine.

Numerous studies have been undertaken to explore the integration of the EFGT cycle with biomass as an energy source for electricity generation [9,10,11]. Despite its benefits, the EFGT cycle often faces criticism for its relatively low energy efficiency, primarily attributed to the biomass’s inferior calorific value [12]. To enhance the efficiency of biomass-powered plants, additional systems for waste heat recovery, such as the steam Rankine cycle (SRC) and organic Rankine cycle (ORC), have been implemented to recover waste heat and convert it into additional power. Research by Soltani et al. [10] on the thermodynamic performance of a biomass gasification integrated EFGT combined cycle demonstrated potential energy and exergy efficiencies of 46.95% and 39.37%, respectively. Mondal et al. [13] undertook an exergoeconomic analysis of a BIGCC system incorporating an EFGT cycle and a supercritical ORC, achieving energy and exergy efficiencies of 40.77% and 36.30%, respectively. Vera et al. [14] evaluated a small-scale power generation setup comprising a downdraft gasifier, an EFGT cycle, and an ORC, demonstrating that the system could attain a net electrical efficiency of 20.7% when employing isopentane as the working fluid for the ORC. Zhang et al. [15] examined a municipal solid waste (MSW) fueled cogeneration system incorporating an EFGT cycle, an SCO_2_ cycle, and a high-temperature organic flash cycle (OFC). They reported the system energy and exergy efficiencies of 75.8% and 41.21% respectively, with a total product cost of 10.2 USD/GJ. Moradi et al. [16] conducted a comparative sensitivity analysis of two micro-scale integrated prime movers based on a GT cycle and an SCO_2_ cycle with bottoming ORC units. The study showed that at full load, the average net electric power output of the SCO_2_ integrated system is about 25% higher than that of the GT system, although it incurs a 75% higher biomass consumption due to lower net electric efficiency. Sharafi laleh et al. [17] assessed the thermodynamic performance of a biomass gasification-based power plant integrated with an EFGT cycle and an SCO_2_ cycle, achieving an energy efficiency of 41.18%.

The combination of biomass energy with multi-generation systems is considered as a strategic approach to boost energy efficiency and satisfy the diverse energy demands of consumers. Advancements in technology have significantly increased the efficiency with which biomass energy is utilized. Biomass-fueled EFGT cycles are supposed to be favorable options for small- to medium-sized multi-generational systems [8,18]. Roy et al. [19] performed techno-economic and environmental analyses of a biomass-based power generation setup integrating a solid oxide fuel cell module (SOFC), an EFGT cycle, and an ORC, with findings indicating potential energy and exergy efficiencies of up to 49.47% and 44.2%, respectively. El-Sattar et al. [20] conducted a thermodynamic study on a combined cooling, heating, and power (CCHP) system including an EFGT cycle, an ORC, and an ARC. They pointed out that toluene is the optimal working fluid for maximizing system thermal efficiency at 43.9%. Roy et al. [21] evaluated a combined power and heating system featuring an EFGT cycle, a biomass gasifier, an SOFC, and a heat recovery steam generator (HRSG), reporting an optimal exergy efficiency and LCOE of 46.58% and 0.0657 USD/kWh, respectively. Zhang et al. [22] analyzed a biomass-fueled cogeneration system, incorporating a gasifier, an EFGT cycle, an SCO_2_ cycle, a Stirling engine, and a DWH. They determined the system’s optimal exergy efficiency to be 46.48% with a total cost rate of 401.4 USD/h. Xu et al. [23] conducted thermodynamic and exergoeconomic analyses on a biomass-fueled multigeneration system, including a syngas production unit, an SRC, a multi-effect desalination (MED) unit, and a solid oxide electrolyzer cell (SOEC). They reported that the optimal exergy efficiency and unit exergy cost could reach 17.64% and 26 USD/GJ respectively. Du et al. [24] analyzed a biomass-driven multigeneration system comprising a gasification unit, a helium GT cycle, a Kalina cycle, a DWH, a refrigeration unit, and a dual-loop OFC. The results indicated that the system could reach an optimal exergy efficiency of 35.57%, a net present value (NPV) of 15.07 M USD, and a payback period of 3.97 years. Yilmaz et al. [25] proposed a biomass-based multigeneration plant with a GT cycle, an SCO_2_ cycle, a multi-stages flash desalination (MSFD) unit, a proton exchange membrane electrolyzer (PEME), and a DWH. They found the energy and exergy efficiencies to be 44.50% and 30.01%, respectively. Zhang et al. [26] proposed a biomass-based multigeneration setup with a GT cycle, an SCO_2_ cycle, a double-effect ARC, a DWH, an ORC, and a reverse osmosis (RO) desalination unit. They concluded that the system could attain an optimal exergy efficiency of 38.54%, along with a sum unit cost of product (SUCP) of 30.8 USD/GJ, and an NPV of 75.17 M USD.

The results of previous research have demonstrated that integrating the EFGT cycle with waste heat recovery systems significantly enhances the efficiency of biomass energy utilization. As a general power generation technology, the SRC has been widely adopted to recover the medium- or high-temperature waste heat. Nonetheless, a considerable amount of energy is released into the environment unutilized during the SRC condensation process. Research has suggested the potential of employing low-temperature condensation heat to drive a single-effect ARC [27]. Liang et al. [28,29] developed a CCP system coupling of an SRC and an ARC to capitalize on the waste heat from a marine engine. They discovered that this SRC–ARC configuration markedly elevates the energy utilization efficiency over the basic SRC, with an 84% increase in exergy efficiency under specific conditions of condensation temperature at 323 K and superheat at 100 K. Ahmadi et al. [30] conducted both thermodynamic and exergoenvironmental evaluations of a GT-based trigeneration system integrated with an SRC and a steam-driven ARC, revealing thermal and exergy efficiencies of 75.5% and 47.5%, respectively. Sahoo et al. [31] thermodynamically evaluated a multi-generation system powered by solar and biomass energies, in which an ARC was driven by the residual heat of the SRC, achieving energy and exergy efficiencies of 49.85% and 20.95%, respectively. Nondy et al. [32] compared the thermodynamic performance of four CCP configurations designed for waste heat recovery from a GT cycle, utilizing SRC and ARC as bottoming cycles. They found that the configuration with two ARCs driven, respectively, by steam and exhaust gas is the most appropriate from the energy and exergy viewpoints. Anvari et al. [33] performed an advanced exergetic and exergoeconomic analysis of a CCHP system consisting of a GT cycle, a dual pressure HRSG, and an ARC driven by the low-pressure steam. They identified that nearly 29% of the total exergy destruction and the associated cost rates due to exergy destruction within the system are endogenous-avoidable.

For an enhanced understanding of the current research in the field, several studies related to biomass-based multigeneration system integrated with a GT cycle have been systematically organized in Table 1. The review of the above studies indicates that many researchers have proposed various biomass-based multi-generation systems with the aim of increasing energy utilization efficiency and reducing environmental impact. It is also suggested that the SRC–ARC combined cycles help to further utilize the waste heat and improve the thermodynamic performance. According to the literature review, devising a high-efficient combined cooling and power system based on biomass gasification combined with EFGT cycle has not been extensively investigated up to now. In addition, the coupling of SRC and ARC integrated with the biomass gasification has seldom been considered in the literature. Considering these motivations, this study aims to provide a comprehensive evaluation of a novel CCP system including an EFGT, an SRC, an ARC, and an ORC based on cascade utilization of high-temperature waste heat from syngas combustion. It can be expected that the proposed scheme has great potential to achieve a noticeable energy efficiency compared to the available literature due to better integration of bottoming sub-cycles. The main objectives and contributions of this work can be summarized as follows:

(1) Introduction of a novel biomass gasification-based CCP system to enhance the energy utilization efficiency, alongside the development of comprehensive mathematical models to assess system performance from thermodynamic and exergoeconomic perspectives.
(2) Examination of the influence of critical operational parameters on the performance criteria.
(3) Optimization of the system to determine the optimal operational conditions that maximize exergy efficiency while minimizing the LCOE.

## 2. System Description

**Figure 1:**
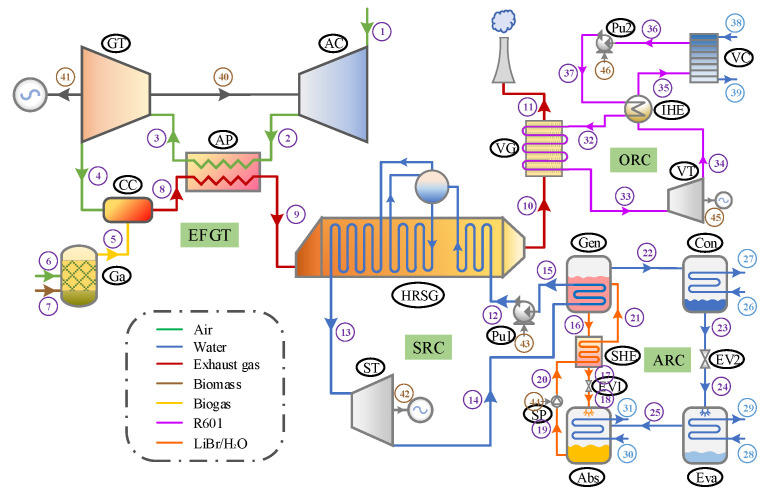
Schematic diagram of the proposed CCP system.

Figure 1 illustrates the configuration of the proposed CCP system, which is fed by biomass and encompasses a biomass gasifier, an EFGT, an SRC, an ARC, and an ORC. Within the EFGT cycle, key components include an air compressor (AC), an air preheater (AP), a combustion chamber (CC), and a gas turbine (GT). Ambient air (state 1) undergoes compression in the AC, and this compressed air (state 2) is then heated by the flue gases (state 8) in the AP. The high-temperature air (state 3) expands through the GT, driving the generator to produce electricity. Subsequently, the exhaust air (state 4) flows into the CC, where it reacts with syngas (state 5) from the biomass gasifier (Ga). After rejecting heat to the compressed air in the AP, the flue gas (state 9) is directed through a heat recovery steam generator (HRSG) and a vapor generator (VG), successively activating the bottoming SRC and ORC.

Within the SRC, the pressurized water (state 12) absorbs heat to be converted into superheated vapor (state 13), which is then expanded in the steam turbine (ST) to generate electricity. The resulting exhaust (state 14) serves as the thermal source for a single-effect LiBr-H_2_O ARC. In the ARC generator (Gen), the dilute solution (state 21) is heated, separating into a concentrated solution (state 16) and refrigerant vapor (state 22). This concentrated solution is then routed through a solution heat exchanger (SHE), warming the returning dilute solution (state 20) back to the generator. Concurrently, the refrigerant vapor condenses in the condenser (Con), and the resulting saturated liquid (state 23) moves to the evaporator (Eva) via an expansion valve (EV2). After absorbing heat in the evaporator, the vaporized refrigerant (state 25) is absorbed by the concentrated solution (state 18) and cooled by the water in the absorber (abs), producing a dilute solution (state 19) that is cycled back through the SHE to the generator.

The exhaust gas is introduced to the bottoming ORC to further exploit its residual thermal energy. The ORC mainly includes the following components: vapor generator (VG), vapor turbine (VT), internal heat exchanger (IHE), condenser (VC), and pump (Pu2). High-pressure vapor (state 33) generated in the VG drives the VT to produce power. The IHE facilitates preheating of the organic liquid (state 37) by the low-pressure vapor (state 34) exiting the VT. This vapor (state 35) condenses into a saturated liquid (state 36) in the VC, releasing heat to the cooling water, before being recirculated by the pump (Pu2) back to the VG via the IHE.

## 3. Mathematical Modeling

### 3.1. Assumptions

The system under consideration is conceptualized and analyzed under a set of foundational assumptions as follows [34,35,36]:

1. Operation of the system is assumed to be in a steady state;
2. Changes in kinetic and potential energy within the system are considered negligible;
3. The system assumes no heat losses across its various components;
4. Pressure variations across piping systems are overlooked;
5. The composition of ambient air is taken as 21% oxygen and 79% nitrogen by volume;
6. Gas mixtures within the system are treated as ideal gases for the purpose of simulation;
7. Within the ARC, fluid streams exit both the evaporator and condenser in a saturated state, and the output solutions from the generator and absorber reach equilibrium at their specific temperatures and concentrations;
8. For the ORC, the working fluid departs the vapor generator as saturated vapor and exits the condenser as saturated liquid;
9. The performance of compressors, pumps, and turbines is modeled with constant isentropic efficiencies.

### 3.2. Energy Analysis

#### 3.2.1. Biomass Gasifier

This study focuses on an atmospheric downdraft gasifier, utilizing wood chips as fuel and air as the gasifying agent. The composition and higher heating value (HHV) of wood is presented in Table 2. The gasification occurs at high temperatures and involves several key stages: drying, pyrolysis, oxidation, and reduction [37]. To estimate the syngas composition, an equilibrium model is employed, demonstrating a reliable approximation of the gasification process within the downdraft gasifier [38,39]. This model assumes that all chemical reactions are in thermodynamic equilibrium and the pyrolysis products reach equilibrium in the reduction zone prior to exiting the gasifier [40,41]. The comprehensive reaction governing biomass fuel gasification can be succinctly represented as follows [17]:

(1) $${\mathrm{CH}}_{1.44}O_{0.66}{+wH}_{2}{O+n}_{\mathrm{air},1}\left(O_{2}+3.76N_{2}\right)\to n_{1}H_{2}{+n}_{2}{\mathrm{CO}+n}_{3}{\mathrm{CO}}_{2}{+n}_{4}H_{2}{O+n}_{5}{\mathrm{CH}}_{4}{+n}_{6}N_{2}$$

where CH_a_O_b_N_c_ represents the chemical composition of the biomass, *w* indicates the moisture content of the biomass, and $n_{\mathrm{air},1}$ signifies the kilomoles of oxygen from air involved in the reaction. The coefficients *n*_1_ to *n*_6_ denote the kilomoles of the product constituents.

The moisture content in the biomass is typically quantified by its mass-based moisture content (*MC*), calculated using the formula below [15]:

(2) $$w=\frac{{\mathrm{MC}\times \mathrm{MW}}_{\mathrm{biomass}}}{{\mathrm{MW}}_{H_{2}O}\times (1-\mathrm{MC})}$$

where ${\mathrm{MW}}_{\mathrm{biomass}}$ and ${\mathrm{MW}}_{H_{2}O}$ refer to the molecular weights of the biomass and water, respectively.

Key reactions occurring during gasification include methane formation and water-gas shift reactions, with the equilibrium constants for these reactions provided as follows [15,23,24]:

(3) $${C+\mathrm{CO}}_{2}⇌2\mathrm{CO}$$

(4) $${\mathrm{CO}+H}_{2}O⇌{\mathrm{CO}}_{2}{+H}_{2}$$

The equilibrium constants for these reactions are denoted as follows [15,23,24]:

(5) $$K_{1}=\frac{n_{5}}{{n_{1}}^{2}}{\left(\frac{P_{\mathrm{Ga}}/P_{\mathrm{ref}}}{n_{\mathrm{tot}}}\right)}^{-1}$$

(6) $$K_{2}=\frac{n_{1}n_{3}}{n_{2}n_{4}}{\left(\frac{P_{\mathrm{Ga}}/P_{\mathrm{ref}}}{n_{\mathrm{tot}}}\right)}^{0}$$

where *P*_Ga_ is the pressure during gasification. *K*_1_ and *K*_2_ are derived from the Gibbs free energy changes associated with each reaction, calculated by [15,23,24]:

(7) $${\mathrm{In}K}_{1}=-\frac{{\Delta G}_{1}^{0}}{{\bar{R}T}_{\mathrm{Ga}}}$$

(8) $${\mathrm{In}K}_{2}=-\frac{{\Delta G}_{2}^{0}}{{\bar{R}T}_{\mathrm{Ga}}}$$

where $\bar{R}$ is the universal gas constant, $T_{\mathrm{Ga}}$ is the temperature within the gasifier. ${\Delta G}_{1}^{0}$ and ${\Delta G}_{2}^{0}$ are computed using the following equations [15,23,24]:

(9) $${\Delta G}_{1}^{0}=\left({\bar{h}}_{{\mathrm{CH}}_{4}}-T_{\mathrm{Ga}}{\bar{s}}_{{\mathrm{CH}}_{4}}^{0}\right)-2\left({\bar{h}}_{H_{2}}-T_{\mathrm{Ga}}{\bar{s}}_{H_{2}}^{0}\right)$$

(10) $${\Delta G}_{2}^{0}=\left({\bar{h}}_{{\mathrm{CO}}_{2}}-T_{\mathrm{Ga}}{\bar{s}}_{{\mathrm{CO}}_{2}}^{0}\right)+\left({\bar{h}}_{H_{2}}-T_{\mathrm{Ga}}{\bar{s}}_{H_{2}}^{0}\right)-\left({\bar{h}}_{\mathrm{CO}}-T_{\mathrm{Ga}}{\bar{s}}_{\mathrm{CO}}^{0}\right)-\left({\bar{h}}_{H_{2}O}-T_{\mathrm{Ga}}{\bar{s}}_{H_{2}O}^{0}\right)$$

Under the assumption of no heat loss in the gasifier, the energy balance equation governing the gasification process is outlined as follows [15,23,24]:

(11) $${\bar{h}}_{f-\mathrm{biomass}}^{0}{+w\bar{h}}_{{f-H}_{2}O}^{0}{+n}_{\mathrm{air},1}{\bar{h}}_{\mathrm{air},1}{=n}_{1}\left({\bar{h}}_{{f-H}_{2}}^{0}{+\Delta \bar{h}}_{H_{2}}\right){+n}_{2}\left({\bar{h}}_{f-\mathrm{CO}}^{0}{+\Delta \bar{h}}_{\mathrm{CO}}\right){+n}_{3}\left({\bar{h}}_{{f-\mathrm{CO}}_{2}}^{0}{+\Delta \bar{h}}_{{\mathrm{CO}}_{2}}\right)\;{+n}_{4}\left({\bar{h}}_{{f-H}_{2O}}^{0}{+\Delta \bar{h}}_{H_{2}O}\right)\;{+n}_{5}\left({\bar{h}}_{{f-\mathrm{CH}}_{4}}^{0}{+\Delta \bar{h}}_{{\mathrm{CH}}_{4}}\right)\;{+n}_{6}\left({\bar{h}}_{{f-N}_{2}}^{0}{+\Delta \bar{h}}_{N_{2}}\right)$$

where ${\bar{h}}_{f-j}^{0}$ corresponds to the formation enthalpy of the *j*th component, ${\Delta \bar{h}}_{j}$ represents the variance in specific enthalpy of the *j*th component at the gasification temperature relative to the reference temperature *T*_0_.

#### 3.2.2. Combustion Chamber

Within the combustion chamber, syngas generated from the gasification process undergoes combustion by reacting with the oxygen in the air supplied by the gas turbine. The chemical reaction is shown as follows under the assumption of complete combustion taking place [15]:

(12) $$n_{1}H_{2}{+n}_{2}{\mathrm{CO}+n}_{3}{\mathrm{CO}}_{2}{+n}_{4}H_{2}{O+n}_{5}{\mathrm{CH}}_{4}{+n}_{6}N_{2}{+n}_{\mathrm{air},2}\left(O_{2}+3.76N_{2}\right)\to \;n_{7}{\mathrm{CO}}_{2}{+n}_{8}H_{2}{O+n}_{9}O_{2}{+n}_{10}N_{2}$$

where $n_{\mathrm{air},2}$ denotes the kilomoles of the oxygen entering the combustion chamber.

For an adiabatic combustion scenario, the energy equation governing the combustion chamber is formulated as follows [15]:

(13) $$\underset{j}{\sum }n_{j}{\left({\bar{h}}_{f-j}^{0}+\Delta \bar{h}\right)}_{\mathrm{air}\;}+\underset{j}{\sum }n_{j}{\left({\bar{h}}_{f-j}^{0}+\Delta \bar{h}\right)}_{\mathrm{syngas}}=\underset{j}{\sum }n_{j}{\left({\bar{h}}_{f-j}^{0}+\Delta \bar{h}\right)}_{\mathrm{exh}}$$

where $n_{j}$ indicates the kilomoles of the *j*th component in the air, syngas, and exhaust gas.

#### 3.2.3. Other System Components

At steady-state operation, the system is governed by equations representing mass balance, energy balance, and concentration balance for each component, expressed as [26,36]:

(14) $$\sum {\dot{m}}_{\mathrm{in}}=\sum {\dot{m}}_{\mathrm{out}}$$

(15) $$\dot{Q}+\sum {\dot{m}}_{\mathrm{in}}h_{\mathrm{in}}=\dot{W}+\sum {\dot{m}}_{\mathrm{out}}h_{\mathrm{out}}$$

(16) $$\sum {\dot{m}}_{\mathrm{in}}X_{\mathrm{in}}=\sum {\dot{m}}_{\mathrm{out}}X_{\mathrm{out}}$$

where *X* symbolizes the mass concentration of LiBr in the solution.

The equations related to mass and energy balances within the system’s components are detailed in Table 3.

### 3.3. Exergy Analysis

The exergy rate balance equation is formulated as follows [42]:

(17) $${\dot{\mathrm{Ex}}}_{F}={\dot{\mathrm{Ex}}}_{P}+{\dot{\mathrm{Ex}}}_{D}+{\dot{\mathrm{Ex}}}_{L}$$

where ${\dot{\mathrm{Ex}}}_{F}$ and ${\dot{\mathrm{Ex}}}_{P}$ reflect the input fuel rate and output product rate, respectively. ${\dot{\mathrm{Ex}}}_{D}$ and ${\dot{\mathrm{Ex}}}_{L}$ correspond to the exergy destruction rate and exergy loss rate, respectively. The detailed exergy balance equations for the system’s components are provided in Table 4.

Disregarding kinetic and potential exergies allows for categorizing the specific exergy of a flow into its physical and chemical components [42]:

(18) $${\mathrm{ex}}_{i}{=\mathrm{ex}}_{i}^{\mathrm{ph}}{+\mathrm{ex}}_{i}^{\mathrm{ch}}$$

The physical exergy is defined as [42]:

(19) $${\mathrm{ex}}_{i}^{\mathrm{ph}}{=h}_{i}-h_{0}-T_{0}\left(s_{i}-s_{0}\right)$$

For an ideal gas mixture, chemical exergy is expressed as [42]:

(20) $${\mathrm{ex}}_{i}^{\mathrm{ch}}=\underset{i}{\sum }x_{i}{\mathrm{ex}}_{0,i}^{\mathrm{ch}}{+\bar{R}T}_{0}\underset{i}{\sum }x_{i}{\mathrm{In}x}_{i}$$

where $x_{i}$ denotes the molar fraction of the *i*th component, ${\mathrm{ex}}_{0,i}^{\mathrm{ch}}$ represents the standard chemical exergy.

The specific chemical exergy of biomass is determined based on its lower heating value (LHV) [10,23,26]:

(21) $$e_{\mathrm{biomass}}^{\mathrm{ch}}{=\psi \mathrm{LHV}}_{\mathrm{biomass}}$$

The coefficient ψ is calculated considering the mass fractions of oxygen (*M*_O_), carbon (*M*_C_), and hydrogen (*M*_H_) within the biomass [10,23,26]:

(22) $$\;\psi =\frac{1.044+0.016\frac{M_{H}}{M_{C}}-0.34493\frac{M_{O}}{M_{C}}\left(1+0.0531\frac{M_{H}}{M_{C}}\right)}{1-0.4124\frac{M_{O}}{M_{C}}}$$

The efficiency in terms of exergy for *k*th component is defined as [42]:

(23) $$\eta_{\mathrm{ex},k}=\frac{{\dot{\mathrm{Ex}}}_{P,k}}{{\dot{\mathrm{Ex}}}_{F,k}}$$

The exergy destruction ratio of the *k*th component is conceptualized as the proportion of that component’s exergy destruction relative to the overall exergy destruction within the system [42]:

(24) $$y_{D,k}=\frac{{\dot{\mathrm{Ex}}}_{D,k}}{{\dot{\mathrm{Ex}}}_{D,\mathrm{tot}}}$$

### 3.4. Exergoeconomic Analysis

Exergoeconomic analysis integrates exergy assessment with economic theories to elucidate the cost generation mechanism and calculate the cost associated with each unit of exergy of the product. The cost balance for *k*th component is formulated as below [26,42]:

(25) $$\sum {\dot{C}}_{\mathrm{in},k}+\sum {\dot{C}}_{q,k}{+\dot{Z}}_{k}=\sum {\dot{C}}_{\mathrm{out},k}{+\dot{C}}_{w,k}$$

where ${\dot{C}}_{j}$ denotes the cost rate (USD/h), ${\dot{Z}}_{k}$ signifies the total cost rate encompassing both capital investment and operational and maintenance expenses for the *k*th component. Table 5 outlines the cost balance and supplementary equations for the system’s components.

The cost rate can be written as [26,42]:

(26) $${\dot{C}}_{j}{=c}_{j}{\dot{\mathrm{Ex}}}_{j}$$

where *c* stands for the cost per unit of exergy (USD/GJ).

To translate capital investment of the *k*th component into a cost rate, the equation below is utilized [15]:

(27) $${\dot{Z}}_{k}=\frac{\mathrm{CRF}\times \phi_{r}{\times Z}_{k}}{N}$$

where $\phi_{r}$ represents maintenance factor (1.06), *N* refers to the number of operating hours annually (7000), $Z_{k}$ indicates the capital cost of the *k*th component. The capital recovery factor (*CRF*) is determined through the formula presented below [15,26]:

(28) $$\mathrm{CRF}=\frac{i_{r}{{(1+i}_{r})}^{n_{t}}}{{{(1+i}_{r})}^{n_{t}}-1}$$

where *i_r_* denotes the annual interest rate (15%), *n_t_* is the lifetime of the system (20 years).

The average unit cost of the fuel ($c_{F,k}$), unit cost of the product ($c_{P,k}$), and cost of exergy destruction (${\dot{C}}_{D,k}$) for the *k*th component are defined, respectively, in subsequent equations [42]:

(29) $$c_{F,k}=\frac{{\dot{C}}_{F,k}}{{\dot{\mathrm{Ex}}}_{F,k}}$$

(30) $$c_{P,k}=\frac{{\dot{C}}_{P,k}}{{\dot{\mathrm{Ex}}}_{P,k}}$$

(31) $${\dot{C}}_{D,k}{=c}_{F,k}{\dot{\mathrm{Ex}}}_{D,k}$$

The relative cost difference (*r_k_*) and exergoeconomic factor (*f_k_*) for the *k*th component are characterized by the following definitions [42]:

(32) $$r_{k}=\frac{c_{P,k}-c_{F,k}}{c_{F,k}}$$

(33) $$f_{k}=\frac{{\;\dot{Z}}_{k}}{{\;\dot{Z}}_{k}{+\dot{C}}_{D,k}}$$

Capital costs for system components are preliminarily calculated using cost functions, which are tabulated in Table 6. These costs, based on reference year values ($Z_{\mathrm{ref}}$), require adjustment to current values ($Z_{\mathrm{PY}}$) employing the chemical engineering plant cost index (CEPCI) [15]:

(34) $$Z_{\mathrm{PY}}{=Z}_{\mathrm{ref}}\times \frac{{\mathrm{CEPCI}}_{\mathrm{PY}}}{{\mathrm{CEPCI}}_{\mathrm{ref}}}$$

Capital cost estimations for heat exchangers necessitate initial determination of heat transfer areas (*A_k_*), calculated by the following equation [15,26]:

(35) $$A_{k}=\frac{{\dot{Q}}_{k}}{U_{k}\Delta T_{\mathrm{lm},k}}$$

where $\Delta T_{\mathrm{lm},k}$ denotes the logarithmic mean temperature difference, *U_k_* represents the heat transfer coefficient. The determination of heat transfer coefficients for heat exchangers is elaborated in Appendix B.

### 3.5. Overall Performance Assessment

The thermal efficiency of the proposed system is calculated as:

(36) $$\eta_{\mathrm{th}}=\frac{{\dot{W}}_{\mathrm{GT}}{+\dot{W}}_{\mathrm{ST}}{+\dot{W}}_{\mathrm{VT}}-{\dot{W}}_{\mathrm{AC}}-{\dot{W}}_{\mathrm{pu}1}-{\dot{W}}_{\mathrm{pu}2}{+\dot{Q}}_{\mathrm{eva}}}{{\dot{m}}_{\mathrm{biomass}}{\mathrm{LHV}}_{\mathrm{biomass}}}$$

The exergy efficiency of the proposed system is computed by:

(37) $$\eta_{\mathrm{ex}}=\frac{{\dot{W}}_{\mathrm{GT}}{+\dot{W}}_{\mathrm{ST}}{+\dot{W}}_{\mathrm{VT}}-{\dot{W}}_{\mathrm{AC}}-{\dot{W}}_{\mathrm{pu}1}-{\dot{W}}_{\mathrm{pu}2}+\left({\dot{\mathrm{Ex}}}_{29}-{\dot{\mathrm{Ex}}}_{28}\right)}{{\dot{\mathrm{Ex}}}_{1}+{\dot{\mathrm{Ex}}}_{6}+{\dot{\mathrm{Ex}}}_{7}}$$

The levelized cost of exergy (LCOE) is adopted as the criterion for evaluating the exergoeconomic performance of the system, formally defined as:

(38) $${\mathrm{LCOE}}_{\mathrm{sys}}=\frac{{\dot{C}}_{41}{+\dot{C}}_{42}{+\dot{C}}_{45}-{\dot{C}}_{43}-{\dot{C}}_{44}-{\dot{C}}_{46}+{\dot{C}}_{29}}{{\dot{W}}_{\mathrm{GT}}{+\dot{W}}_{\mathrm{ST}}{+\dot{W}}_{\mathrm{VT}}-{\dot{W}}_{\mathrm{AC}}-{\dot{W}}_{\mathrm{pu}1}-{\dot{W}}_{\mathrm{pu}2}+{\dot{\mathrm{Ex}}}_{29}}$$

### 3.6. Multi-Objective Optimization

Optimization plays a critical role in enhancing the performance of energy system designs, particularly in thermal systems where design objectives often present conflicting requirements, making it challenging to achieve an optimal solution that meets all criteria simultaneously. To navigate these complexities, multi-objective optimization techniques are frequently employed. This approach involves defining objective functions, decision variables, and their respective boundaries, which can be described as follows [45]:

(39) $$\min\;F(X)={\left[f_{1}{(X),\;f}_{2}{(X),\ldots ,\;f}_{k}\left(X\right)\right]}^{T}$$

subject to

(40) $$g_{i}(X)\;\leq \;0,\;i=1,\ldots ,\;m$$

(41) $$h_{j}(X)=0,\;j=1,\ldots ,\;n$$

(42) $$X_{k,\min\;}\leq {\;X}_{k}\;\leq {\;X}_{k,\max}$$

where *X*, *F*(*X*), and *f*(*X*) indicate the vectors of decision variables, multi-objective function, and single-objective function, respectively; $g_{i}\left(X\right)$ and $h_{i}\left(X\right)$ represent the inequality and equality constraints, respectively; $X_{k,\min\;}$ and $X_{k,\max}$ stand for the bottom and top bounds of the *k*th decision variables, respectively.

In the current research, the genetic algorithm (GA) is utilized to address the multi-objective optimization issue. This technique begins by creating an initial population of solution candidates, which undergoes evolution through random selection from the existing population. This population is then evolved using a series of operations including selection, mutation, crossover, and inheritance. Over successive generations, the most favorable solutions emerge and are compiled into a Pareto frontier, with each point on this frontier representing a viable optimal solution [46]. The ultimate solution is identified using TOPSIS (Technique for Order of Preference by Similarity to Ideal Solution) decision making [47]. The TOPSIS method introduces two hypothetical solutions: the “ideal point”, which signifies the optimal values for each objective, and the “non-ideal point”, representing the worst values. The solution that lies nearest to the ideal point and furthest from the non-ideal point is adjudged the ultimate optimal solution. The methodology for constructing the decision matrix and computing the distance of each solution to the ideal and non-ideal points is detailed as follows [23,26]:

(43) $$F_{\mathrm{ij}}=\frac{x_{\mathrm{ij}}}{\sqrt{\sum_{i=1}^{m}x_{\mathrm{ij}}^{2}}}$$

(44) $$D_{i+}=\sqrt{\overset{n}{\underset{j=1}{\sum }}{\left(F_{\mathrm{ij}}{-F}_{\mathrm{ij}}^{\mathrm{ideal}}\right)}^{2}}$$

(45) $$D_{i-}=\sqrt{\overset{n}{\underset{j=1}{\sum }}{\left(F_{\mathrm{ij}}{-F}_{\mathrm{ij}}^{\mathrm{non}-\mathrm{ideal}}\right)}^{2}}$$

The relative closeness is defined as:

(46) $${\mathrm{Cl}}_{i}=\frac{D_{i-}}{D_{i+}+D_{i-}}$$

Finally, the solution with maximum *Cl_i_* is considered as the desired final solution.

## 4. Results and Discussion

### 4.1. Model Validation

To validate the mathematical models applied to the proposed system, this study compares simulation outcomes for various components, including the EFGT, biomass gasifier, ORC, and ARC, against findings reported in prior research. Computational models are constructed utilizing MATLAB R2018b software for simulation purposes, and the thermophysical properties of the working fluids are sourced from the REFPROP 9.0 database. The comparison, detailed in Table 7, Table 8, Table 9 and Table 10, reveals a satisfactory concordance between the results of this study and those documented in existing literature.

### 4.2. Base Case Results

Table A1 outlines the base case input parameters for these subsystems, which enable the derivation of simulation outcomes by solving the equations previously described. The characteristics of each fluid stream, encompassing both thermodynamic and economic aspects, are summarized in Table A2. Table A3 details the distribution of exergy and exergoeconomic parameters across the system’s components. Notably, the steam turbine exhibits the highest capital cost rate, succeeded by the air preheater and air compressor. In terms of exergy efficiency, the gas turbine, air preheater, and air compressor exhibit superior performance, whereas the absorber in ARC displays the lowest efficiency.

**Figure 2:**
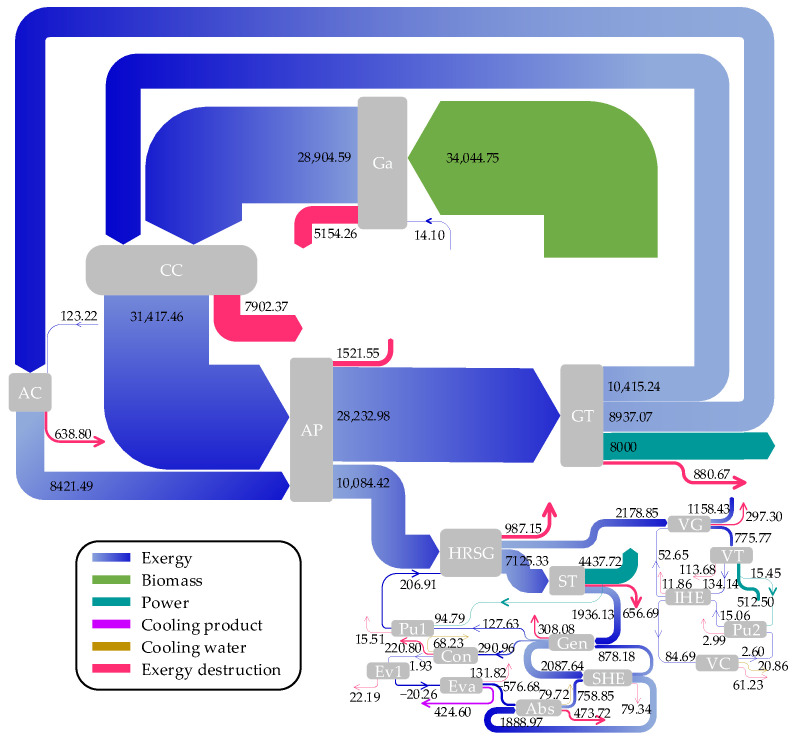
Exergy flow diagram of the system.

**Figure 3:**
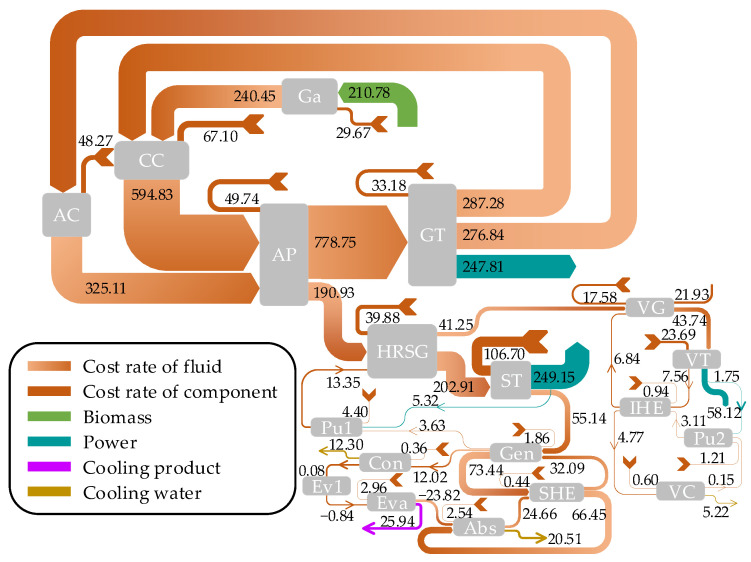
Cost rate flow diagram of the system.

Figure 2 and Figure 3 depict the Sankey diagrams for the exergy and cost rate flows of the proposed system, respectively. As shown in Figure 2, the input exergy rate from wood biomass fuel surpasses other sources, amounting to 34,044.75 kW. The combustion chamber is identified as the main contributor to the system’s total exergy destruction. In Figure 3, the air outlet of the air preheater exhibits the highest cost rate within the system at 778.75 USD/h, succeeded by the flue gas outlet of the combustion chamber at 594.83 USD/h.

Thermodynamic and exergoeconomic analyses of the system, as summarized in Table 11, demonstrate the system’s capability to generate a net power output of 12,950.2 kW and a cooling capacity of 7738.4 kW. Additionally, the system achieves total energy and exergy efficiencies of 70.67% and 39.13%, respectively. The analysis also shows a disparity in the unit cost of power production, with the ORC turbine at 31.50 USD/GJ, significantly higher than both the SRC turbine at 15.60 USD/GJ and the gas turbine at 8.60 USD/GJ. Given that the gas turbine and SRC turbine contribute significantly more power than the ORC turbine and that the unit cost of cooling production is lower than that of power generation, the LCOE of the overall system is determined to be 11.67 USD/GJ, reflecting a balanced cost-efficiency ratio.

### 4.3. Parametric Study

#### 4.3.1. Effect of Air Compressor Pressure Ratio on the System Performance

**Figure 4:**
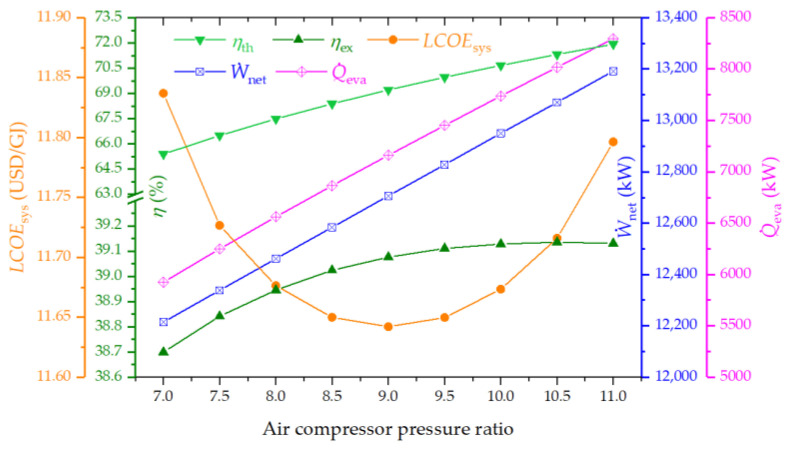
Effect of air compressor pressure ratio on the thermal efficiency, exergy efficiency, LCOE, net power output, and cooling output of the system.

Figure 4 illustrates the influence of air compressor pressure ratio (*PR*_AC_) on the system performance. According to the figure, the thermal efficiency rises considerably as the *PR*_AC_ augments, while the exergy efficiency increases gently and reaches a peak value of 39.1%. The LCOE of the system attains its lowest at 11.64 USD/GJ for a *PR*_AC_ value around 9, beyond which it begins to ascend. Additionally, both the net power and cooling capacity present upward trends as the *PR*_AC_ rises. This trend is attributed to the augmented thermal energy available to the subsequent cycles, driven by the elevation in flue gas temperature at the air preheater exit under a constant CETD. Despite a slight rise in biomass consumption, the total energy output’s augmentation surpasses the increase in biomass input, thus elevating thermal efficiency.

#### 4.3.2. Effect of Gas Turbine Inlet Temperature on the System Performance

**Figure 5:**
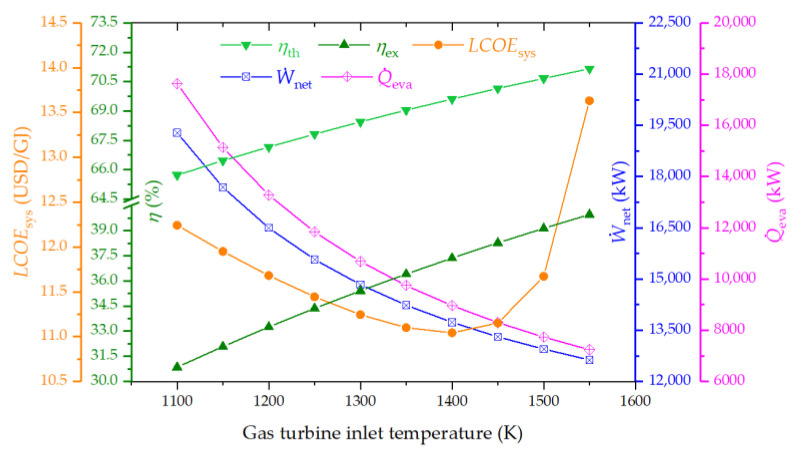
Effect of gas turbine inlet temperature on the thermal efficiency, exergy efficiency, LCOE, net power output, and cooling output of the proposed system.

Figure 5 examines the impact of gas turbine inlet temperature (GTIT) on system performance. This figure reveals that both thermal and exergy efficiencies improve with an ascending GTIT, whereas net power and cooling capacities experience a marked decrease. The LCOE of the system attains its lowest point at a GTIT of approximately 1400 K. The rationale behind these observations lies in the augmented enthalpy difference across the gas turbine as GTIT increases, which in turn significantly reduces the mass flow rates of air and flue gas to maintain a constant power output of EFGT. Consequently, the supply of thermal heat to the subsequent cycles diminishes, leading to the decline of net power and cooling outputs. However, the decrease in biomass consumption, coupled with unchanged efficiencies of the bottoming cycles, contributes to overall increases in both thermal and exergy efficiencies. Nonetheless, the increment in thermal and exergy efficiencies of the EFGT cycle, due to reduced biomass fuel consumption, contributes to an overall increase in the system’s efficiencies, as the efficiencies of bottoming cycles remain unchanged.

#### 4.3.3. Effect of Pinch Point Temperature Difference in HRSG on the System Performance

**Figure 6:**
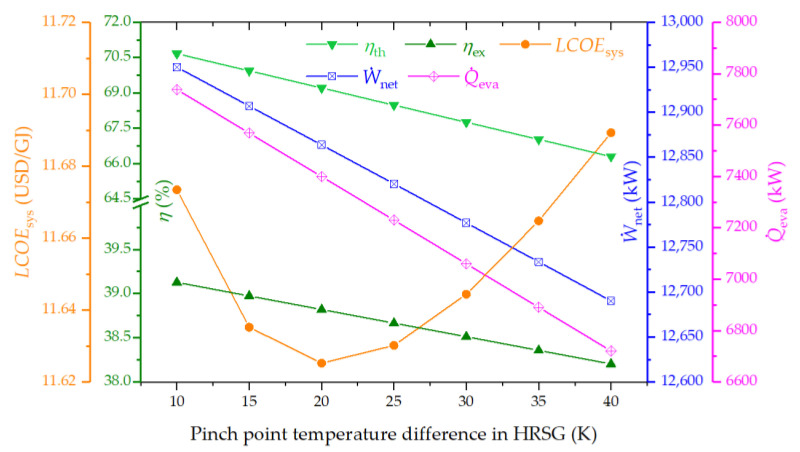
Effect of pinch point temperature difference in HRSG on the thermal efficiency, exergy efficiency, LCOE, net power output, and cooling output of the proposed system.

Figure 6 displays the impact of pinch point temperature difference in HRSG on the system performance. It is observed that elevating the pinch point temperature difference leads to declines in both thermal and exergy efficiencies, alongside reductions in net power and cooling capacity. Conversely, the LCOE initially decreases, reaching a minimum, before it starts to ascend. This trend can be attributed to the widened temperature gap between the high-temperature flue gases and the working fluid in the HRSG, which amplifies exergy destruction and diminishes the thermal energy supplied to the SRC. Consequently, this reduction in heat transfer causes net power and cooling outputs to decline, adversely affecting both thermal and exergy efficiencies.

#### 4.3.4. Effect of Steam Turbine Inlet Pressure on the System Performance

**Figure 7:**
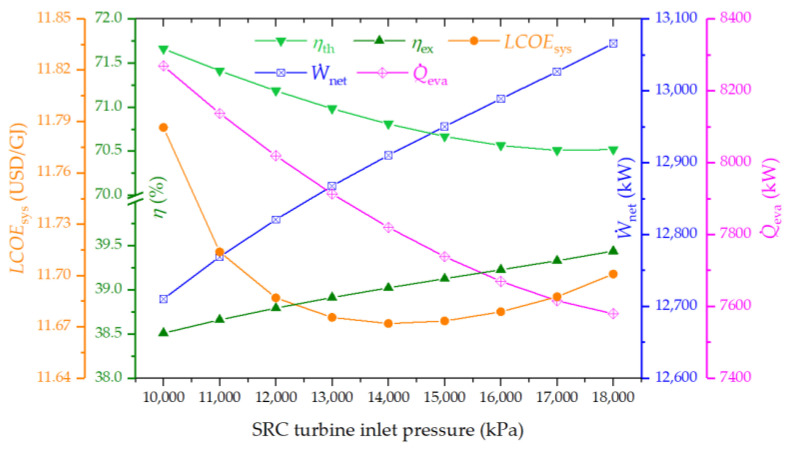
Effect of steam turbine inlet pressure on the thermal efficiency, exergy efficiency, LCOE, net power output, and cooling output of the proposed system.

The impact of the steam turbine inlet pressure (STIP) on system performance is illustrated in Figure 7. It is observed that exergy efficiency enhances with a rise in STIP, whereas thermal efficiency exhibits a gradual decline. The LCOE demonstrates a decrease to a minimum point, subsequently increasing. Additionally, an increase in STIP leads to a boost in net power output due to the enhanced efficiency of the SRC. Conversely, cooling capacity experiences a downturn, attributed to a decreased availability of condensation heat for the ARC. This results in an improvement in exergy efficiency, as the generation of electricity, which is of a higher quality, outweighs the cooling production. The overall thermal efficiency of the system is thus a function of the combined outputs of power and cooling capacity.

#### 4.3.5. Effect of SRC Condenser Temperature on the System Performance

**Figure 8:**
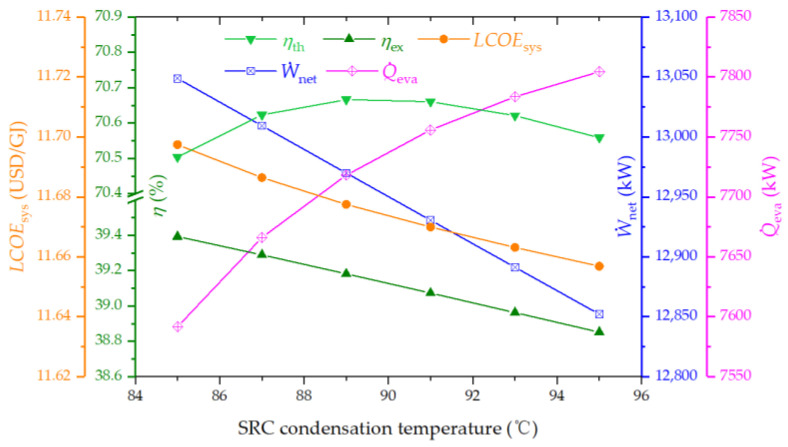
Effect of SRC condenser temperature on the thermal efficiency, exergy efficiency, LCOE, net power output, and cooling output of the proposed system.

Figure 8 depicts the relationship between the SRC condenser temperature and its impact on system metrics. An inverse relationship is noted between the SRC condenser temperature and both the LCOE and exergy efficiency, while thermal efficiency initially rises before showing a decline. This behavior is attributable to several factors. An elevation in the SRC condenser temperature leads to a reduction in net power due to a diminished SRC efficiency. Concurrently, cooling capacity experiences a boost owing to the enhanced COP and increased thermal energy supply to the ARC. Therefore, the variation in thermal efficiency is influenced by the cumulative effect on output power and cooling capacity, whereas the exergy efficiency witnesses a downturn primarily because the reduction in net power has a more pronounced impact than the increase in cooling capacity.

#### 4.3.6. Effect of ORC Turbine Inlet Pressure on the System Performance

**Figure 9:**
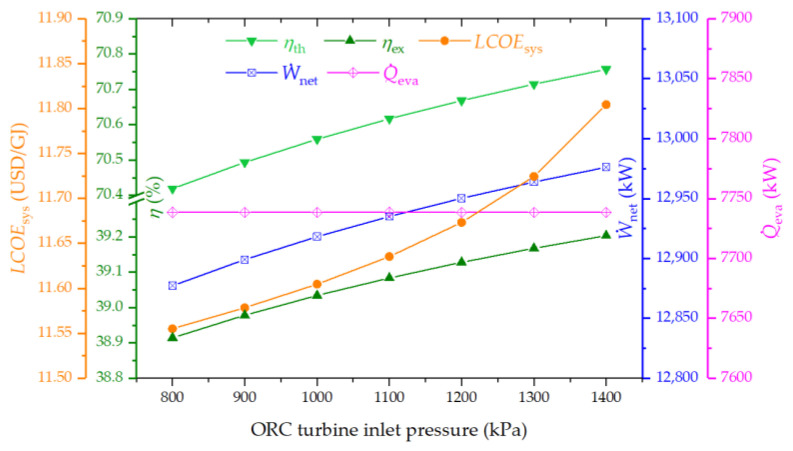
Effect of ORC turbine inlet pressure on the thermal efficiency, exergy efficiency, LCOE, net power output, and cooling output of the proposed system.

The influence of ORC turbine inlet pressure on system performance is sketched in Figure 9. This figure reveals that thermal and exergy efficiencies, LCOE, and output power all experience marginal improvements with the elevation of ORC turbine inlet pressure. Notably, changes in ORC turbine inlet pressure do not affect the performance of the topping cycles. The ORC efficiency improves with higher turbine inlet pressure, facilitating an increase in generated power. Nevertheless, given that the contribution of power from the ORC to the overall system is relatively modest, its impact on the overall system performance is minimal.

### 4.4. Optimization Results

**Figure 10:**
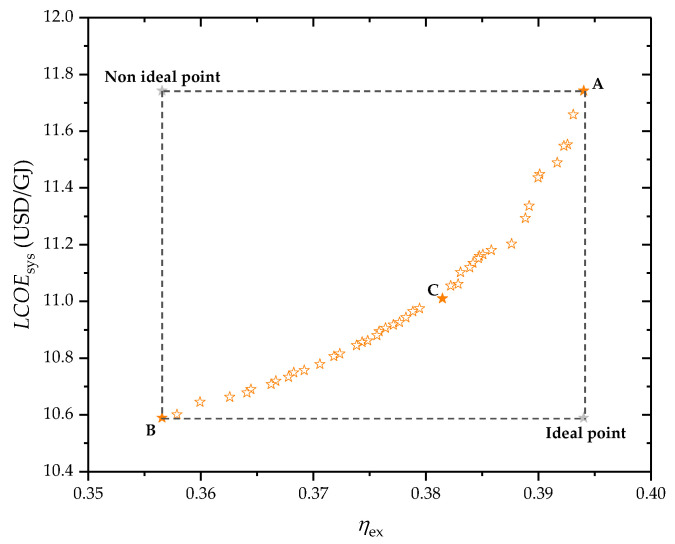
Distribution of the optimal points on the Pareto frontier.

In examining the system performance from both thermodynamic and economic perspectives, the study adopts exergy efficiency and LCOE as its main performance indicators. Through detailed parametric scrutiny, essential operational parameters are delineated as decision-making variables, with their respective ranges provided in Table 12. Utilizing MATLAB R2018b software, a specialized algorithm is created to implement the GA method aimed at optimizing the two objectives. The resulting optimal solutions are depicted as a scattered set across the Pareto frontier in Figure 10, where each marker denotes a potentially optimal configuration, revealing the inherent trade-off between the objectives. Optimal thermodynamic efficiency is achieved at point A, characterized by an exergy efficiency peak of 39.40%, whereas the most favorable economic outcome is observed at point B, showcasing the lowest LCOE at 10.59 USD/GJ. Given this context, the TOPSIS method is employed to determine the ultimate optimal point on the Pareto front, which is identified at point C, balancing an exergy efficiency of 38.15% with an LCOE of 11.01 USD/GJ. The objective function values and the decision variables for points A, B, and C on the Pareto frontier are detailed in Table 13.

### 4.5. Comprative Study

As a final step in presenting the results, a comparison with previously published data is conducted. Under identical operating conditions of the EFGT cycle ($\eta_{\mathrm{is},\mathrm{AC}}=0.87$, $\eta_{\mathrm{is},\mathrm{GT}}=0.89$), the energy and exergy efficiencies as well as cost of products are compared in Table 14. According to Table 14, the designed plant exhibits moderate thermodynamic performance and slightly inferior economic characteristics when assessed against various other systems. When contrasted with the findings from Ref. [22], the current system demonstrates superior energy and exergy efficiencies under the base case conditions. However, in comparison to Ref. [15], it records slightly lower efficiencies under the same simulation conditions. Additionally, the cost of products is marginally higher than those in Refs. [15,22], which is primarily due to the structural design of the system and the methodology used for cost calculation. The systems referenced in Refs. [15,22] are configured to generate both power and heating load, whereas the present study incorporates an ARC to provide a cooling load. Moreover, in Refs. [15,22], municipal solid waste is employed as biomass fuel, in contrast to the wood used in this study, leading to different conversion efficiencies and syngas compositions during the biomass gasification process. Relative to Ref. [17], the system in this study exhibits significantly higher thermal efficiency. This improvement is attributed to the use of additional bottoming cycles for recovering heat from combustion gases generated by biomass-based fuels.

## 5. Conclusions

This research introduces an innovative combined cooling and power system, integrating an EFGT, an SRC, an ORC, and an ARC to enhance biomass energy utilization, and its performance is evaluated from the thermodynamic and exergoeconomic perspectives. A thorough parametric study is performed to ascertain the impact of various design parameters on system performance, while multi-objective optimization focuses on maximizing exergy efficiency and minimizing the LCOE. The main conclusions can be drawn as follows:

- For the baseline scenario, the system exhibits a thermal efficiency of 70.67%, an exergy efficiency of 39.13%, and an LCOE of 11.67 USD/GJ, alongside generating a net power of 12,950.2 kW and a cooling output of 7738.4 kW.
- Exergy analysis revealed that the highest rate of exergy destruction occurs in the combustion chamber, followed closely by the biomass gasifier. The gas turbine and the absorber demonstrated the best and poorest performances from exergy viewpoint among the system components, respectively.
- The inlet temperature of the gas turbine emerged as a critical factor affecting the system performance. Elevating GTIT significantly boosts both thermal and exergy efficiencies, despite a notable reduction in net power and cooling outputs.
- Superior thermodynamic performance is achieved at a higher air compressor pressure ratio and a gas turbine inlet temperature, or at a lower pinch point temperature difference in the HRSG. Optimizing these parameters also leads to minimized LCOE.
- Under optimal conditions, the CCP system demonstrates a 5.7% reduction in LCOE and a 2.5% decrease in exergy efficiency compared to the baseline scenario, highlighting a trade-off between different optimization criteria. This balance suggests that the optimal solution varies depending on specific engineering applications’ requirements.

Future research could concentrate on the enhancement of integrated energy systems by incorporating additional energy sources or subsystems to expand product diversity and improve system functionality. Efforts should be directed toward minimizing exergy destruction rates and maximizing energy utilization to enhance system efficiency, alongside environmental assessment to evaluate operational sustainability. Subsequent studies should also include comparative analyses of diverse biomass feedstocks in gasifiers, exploration of alternative ORC working fluids, and investigation of advanced power generation technologies such as transcritical ORCs and supercritical CO_2_ Brayton cycles to enhance system performance. Practical feasibility assessments and experimental validations with real-world devices are also imperative for advancing the application of developed systems.

## Figures and Tables

**Table 1:** Overview of recent research on the configurations and evaluations of multi-generation systems based on biomass gasification.

Researcher	Year	Biomass Fuel	Configuration	Analysis	Result
Zhang et al. [15]	2023	municipal solid waste	EFGT, SCO_2_ cycle, OFC	energy, exergy, economic, environmental	energy efficiency of 75.8%, exergy efficiency of 41.21%, net profit of 10.7 M USD, levelized CO_2_ emission of 0.518 t/kWh
Moradi et al. [16]	2023	hazelnut shell	GT cycle, SCO_2_ cycle, ORC	energy	25% higher electric power output of the SCO_2_ integrated system
Sharafi laleh et al. [17]	2024	wood	EFGT, SCO_2_ cycle	energy	energy efficiency of 41.8%
Roy et al. [19]	2019	wood, rice husk, paper	EFGT, SOFC, ORC	energy, exergy, economic, environmental	energy efficiency of 49.47%, exergy efficiency of 44.2%
El-Sattar et al. [20]	2020	bagasse	EFGT, ORC, ARC	energy	thermal efficiency of 43.9%
Roy et al. [21]	2020	sawdust	EFGT, SOFC, HRSG	exergy, economic	exergy efficiency of 46.58%, levelized cost of exergy of 0.0657 USD/kWh
Zhang et al. [22]	2022	paddy husk, paper, wood, municipal solid waste	EFGT, SCO_2_ cycle, Stirling engine, DWH	energy, exergy, exergoeconomic, environmental	exergy efficiency of 46.48%, total cost rate of 401.4 USD/h
Xu et al. [23]	2022	paddy husk, paper, wood, municipal solid waste	SRC, MED unit, SOEC	energy, exergy, exergoeconomic	exergy efficiency of 17.64%, unit exergy cost of 26 USD/GJ
Du et al. [24]	2024	wood	helium GT cycle, Kalina cycle, DWH, refrigeration unit, dual-loop OFC	energy, exergy, economic	exergy efficiency of 35.57%, NPV of 15.07 M USD, payback period of 3.97 years
Yilmaz et al. [25]	2024	pine sawdust	GT cycle, SCO_2_ cycle, MSFD unit, PEME, DWH	energy, exergy, environmental	energy efficiency of 44.50%, exergy efficiency of 30.01%
Zhang et al. [26]	2024	carbohydrate	GT cycle, SCO_2_ cycle, dual-effect ARC, DWH, ORC, RO desalination	energy, exergy, economic	exergy efficiency of 38.54%, SUCP of 30.8 USD/GJ, NPV of 75.17 M USD

**Table 2:** Ultimate analysis and higher heating value of wood [17,22].

Biomass	Mass Percentage on Dry Basis (%)						HHV (kJ/kmol)
	C	H	N	S	O	Ash	
wood	50	6	0	0	44	0	449,568

**Table 3:** Mass and energy balance equations for the system components.

Component	Mass and Energy Balance Equations
Air compressor	${\dot{W}}_{\mathrm{AC}}\;{=\dot{m}}_{1}\left(h_{2}-h_{1}\right)$
	${\;\dot{m}}_{1}\;{=\dot{m}}_{2}$
Air preheater	${\;\dot{m}}_{2}\left(h_{3}-h_{2}\right)\;{=\dot{m}}_{8}\left(h_{8}-h_{9}\right)$
	${\;\dot{m}}_{2}\;{=\dot{m}}_{3}$ $,\;{\;\dot{m}}_{8}\;{=\dot{m}}_{9}$
Gas turbine	${\dot{W}}_{\mathrm{GT}\;}\;{=\dot{m}}_{3}\left(h_{3}-h_{4}\right)$
	${\;\dot{m}}_{3}={\dot{m}}_{4}$
HRSG	${\dot{Q}}_{\mathrm{HRSG}}\;={\dot{m}}_{9}\left(h_{9}-h_{10}\right)\;{=\dot{m}}_{12}\left(h_{13}-h_{12}\right)$
	${\;\dot{m}}_{9}={\dot{m}}_{10}$ $,\;{\;\dot{m}}_{12}={\dot{m}}_{13}$
Steam turbine	${\dot{W}}_{\mathrm{ST}}\;{=\dot{m}}_{13}\left(h_{13}-h_{14}\right)$
	${\;\dot{m}}_{13}={\dot{m}}_{14}$
Pump 1	${\dot{W}}_{\mathrm{pu}1}={\dot{m}}_{12}\left(h_{12}-h_{15}\right)$
	${\;\dot{m}}_{12}\;{=\dot{m}}_{15}$
Generator	${\dot{Q}}_{\mathrm{gen}}\;{=\dot{m}}_{14}\left(h_{14}-h_{15}\right)\;{=\dot{m}}_{16}h_{16}+{\;\dot{m}}_{22}h_{22}-{\;\dot{m}}_{21}h_{21}$
	${\;\dot{m}}_{14}\;{=\dot{m}}_{15}$ $,\;{\;\dot{m}}_{21}\;{=\dot{m}}_{16}+{\;\dot{m}}_{22}$
SHE	${\dot{Q}}_{\mathrm{SHE}}\;={\dot{m}}_{16}\left(h_{16}-h_{17}\right)={\dot{m}}_{20}\left(h_{21}-h_{20}\right)$
	${\;\dot{m}}_{16}\;{=\dot{m}}_{17}$ $,\;{\;\dot{m}}_{20}\;{=\dot{m}}_{21}$
Absorber	${\dot{Q}}_{\mathrm{abs}}={\dot{m}}_{30}\left(h_{31}-h_{30}\right)={\dot{m}}_{18}h_{18}+{\;\dot{m}}_{25}h_{25}-{\;\dot{m}}_{19}h_{19}$
	${\;\dot{m}}_{30}={\dot{m}}_{31}$ $,\;{\;\dot{m}}_{19}={\dot{m}}_{18}+{\;\dot{m}}_{25}$
Solution pump	${\dot{W}}_{\mathrm{SP}}={\dot{m}}_{19}\left(h_{20}-h_{19}\right)$
	${\;\dot{m}}_{19}={\dot{m}}_{20}$
Condenser	${\dot{Q}}_{\mathrm{con}}={\dot{m}}_{22}\left(h_{22}-h_{23}\right)={\dot{m}}_{26}\left(h_{27}-h_{26}\right)$
	${\;\dot{m}}_{22}\;{=\dot{m}}_{23}$ $,\;{\;\dot{m}}_{26}={\dot{m}}_{27}$
Evaporator	${\dot{Q}}_{\mathrm{eva}}={\dot{m}}_{24}\left(h_{25}-h_{24}\right)\;{=\dot{m}}_{28}\left(h_{28}-h_{29}\right)$
	${\;\dot{m}}_{24}={\dot{m}}_{25}$ $,\;{\;\dot{m}}_{28}={\dot{m}}_{29}$
Vapor generator	${\dot{Q}}_{\mathrm{VG}}\;{=\dot{m}}_{10}\left(h_{10}-h_{11}\right)={\dot{m}}_{32}\left(h_{33}-h_{32}\right)$
	${\;\dot{m}}_{10}={\dot{m}}_{11}$ $,\;{\;\dot{m}}_{32}={\dot{m}}_{33}$
Vapor turbine	${\dot{W}}_{\mathrm{VT}}={\dot{m}}_{33}\left(h_{33}-h_{34}\right)$
	${\;\dot{m}}_{33}={\dot{m}}_{34}$
IHE	${\dot{Q}}_{\mathrm{IHE}}\;{=\dot{m}}_{37}\left(h_{32}-h_{37}\right)={\dot{m}}_{34}\left(h_{34}-h_{35}\right)$
	${\;\dot{m}}_{32}\;{=\dot{m}}_{37}$ $,\;{\;\dot{m}}_{34}\;{=\dot{m}}_{35}$
Vapor condenser	${\dot{Q}}_{\mathrm{VC}}={\dot{m}}_{35}\left(h_{35}-h_{36}\right)\;{=\dot{m}}_{38}\left(h_{39}-h_{38}\right)$
	${\;\dot{m}}_{35}={\dot{m}}_{36}$ $,\;{\;\dot{m}}_{38}\;{=\dot{m}}_{39}$
Pump 2	${\dot{W}}_{\mathrm{pu}2}\;{=\dot{m}}_{36}\left(h_{37}-h_{36}\right)$
	${\;\dot{m}}_{36}={\dot{m}}_{37}$

**Table 4:** Exergy balance equations for the system components.

Component	$\mathrm{Exergy}\;\mathrm{of}\;\mathrm{Fuel}\;({\dot{\mathrm{Ex}}}_{F}$)	$\mathrm{Exergy}\;\mathrm{of}\;\mathrm{Product}\;({\dot{\mathrm{Ex}}}_{P}$)	$\mathrm{Exergy}\;\mathrm{Destruction}\;({\dot{\mathrm{Ex}}}_{D}$)
Air compressor	${\dot{\mathrm{Ex}}}_{40}$	${\dot{\mathrm{Ex}}}_{2}-{\dot{\mathrm{Ex}}}_{1}$	${\dot{\mathrm{Ex}}}_{F,\mathrm{AC}}-{\dot{\mathrm{Ex}}}_{P,\mathrm{AC}}$
Air preheater	${\dot{\mathrm{Ex}}}_{8}-{\dot{\mathrm{Ex}}}_{9}$	${\dot{\mathrm{Ex}}}_{3}-{\dot{\mathrm{Ex}}}_{2}$	${\dot{\mathrm{Ex}}}_{F,\mathrm{AP}}-{\dot{\mathrm{Ex}}}_{P,\mathrm{AP}}$
Gas turbine	${\dot{\mathrm{Ex}}}_{3}-{\dot{\mathrm{Ex}}}_{4}$	${\dot{\mathrm{Ex}}}_{40}+{\dot{\mathrm{Ex}}}_{41}$	${\dot{\mathrm{Ex}}}_{F,\mathrm{GT}}-{\dot{\mathrm{Ex}}}_{P,\mathrm{GT}}$
Combustion chamber	${\dot{\mathrm{Ex}}}_{4}+{\dot{\mathrm{Ex}}}_{5}$	${\dot{\mathrm{Ex}}}_{8}$	${\dot{\mathrm{Ex}}}_{F,\mathrm{CC}}-{\dot{\mathrm{Ex}}}_{P,\mathrm{CC}}$
Biomass gasifier	${\dot{\mathrm{Ex}}}_{6}+{\dot{\mathrm{Ex}}}_{7}$	${\dot{\mathrm{Ex}}}_{5}$	${\dot{\mathrm{Ex}}}_{F,\mathrm{Ga}}-{\dot{\mathrm{Ex}}}_{P,\mathrm{Ga}}$
HRSG	${\dot{\mathrm{Ex}}}_{9}-{\dot{\mathrm{Ex}}}_{10}$	${\dot{\mathrm{Ex}}}_{13}-{\dot{\mathrm{Ex}}}_{12}$	${\dot{\mathrm{Ex}}}_{F,\mathrm{HRSG}}-{\dot{\mathrm{Ex}}}_{P,\mathrm{HRSG}}$
Steam turbine	${\dot{\mathrm{Ex}}}_{13}-{\dot{\mathrm{Ex}}}_{14}$	${\dot{\mathrm{Ex}}}_{42}$	${\dot{\mathrm{Ex}}}_{F,\mathrm{ST}}-{\dot{\mathrm{Ex}}}_{P,\mathrm{ST}}$
Pump 1	${\dot{\mathrm{Ex}}}_{43}$	${\dot{\mathrm{Ex}}}_{12}-{\dot{\mathrm{Ex}}}_{15}$	${\dot{\mathrm{Ex}}}_{F,\mathrm{Pu}1}-{\dot{\mathrm{Ex}}}_{P,\mathrm{Pu}1}$
Generator	${\dot{\mathrm{Ex}}}_{14}-{\dot{\mathrm{Ex}}}_{15}$	${\dot{\mathrm{Ex}}}_{16}+{\dot{\mathrm{Ex}}}_{22}-{\dot{\mathrm{Ex}}}_{21}$	${\dot{\mathrm{Ex}}}_{F,\mathrm{gen}}-{\dot{\mathrm{Ex}}}_{P,\mathrm{gen}}$
SHE	${\dot{\mathrm{Ex}}}_{16}-{\dot{\mathrm{Ex}}}_{17}$	${\dot{\mathrm{Ex}}}_{21}-{\dot{\mathrm{Ex}}}_{20}$	${\dot{\mathrm{Ex}}}_{F,\mathrm{SHE}}-{\dot{\mathrm{Ex}}}_{P,\mathrm{SHE}}$
Absorber	${\dot{\mathrm{Ex}}}_{18}+{\dot{\mathrm{Ex}}}_{25}-{\dot{\mathrm{Ex}}}_{19}$	${\dot{\mathrm{Ex}}}_{31}-{\dot{\mathrm{Ex}}}_{30}$	${\dot{\mathrm{Ex}}}_{F,\mathrm{abs}}-{\dot{\mathrm{Ex}}}_{P,\mathrm{abs}}$
Solution pump	${\dot{\mathrm{Ex}}}_{44}$	${\dot{\mathrm{Ex}}}_{20}-{\dot{\mathrm{Ex}}}_{19}$	${\dot{\mathrm{Ex}}}_{F,\mathrm{SP}}-{\dot{\mathrm{Ex}}}_{P,\mathrm{SP}}$
Condenser	${\dot{\mathrm{Ex}}}_{22}-{\dot{\mathrm{Ex}}}_{23}$	${\dot{\mathrm{Ex}}}_{27}-{\dot{\mathrm{Ex}}}_{26}$	${\dot{\mathrm{Ex}}}_{F,\mathrm{con}}-{\dot{\mathrm{Ex}}}_{P,\mathrm{con}}$
Evaporator	${\dot{\mathrm{Ex}}}_{24}-{\dot{\mathrm{Ex}}}_{25}$	${\dot{\mathrm{Ex}}}_{29}-{\dot{\mathrm{Ex}}}_{28}$	${\dot{\mathrm{Ex}}}_{F,\mathrm{eva}}-{\dot{\mathrm{Ex}}}_{P,\mathrm{eva}}$
Vapor generator	${\dot{\mathrm{Ex}}}_{10}-{\dot{\mathrm{Ex}}}_{11}$	${\dot{\mathrm{Ex}}}_{33}-{\dot{\mathrm{Ex}}}_{32}$	${\dot{\mathrm{Ex}}}_{F,\mathrm{VG}}-{\dot{\mathrm{Ex}}}_{P,\mathrm{VG}}$
Vapor turbine	${\dot{\mathrm{Ex}}}_{33}-{\dot{\mathrm{Ex}}}_{34}$	${\dot{\mathrm{Ex}}}_{45}$	${\dot{\mathrm{Ex}}}_{F,\mathrm{VT}}-{\dot{\mathrm{Ex}}}_{P,\mathrm{VT}}$
IHE	${\dot{\mathrm{Ex}}}_{34}-{\dot{\mathrm{Ex}}}_{35}$	${\dot{\mathrm{Ex}}}_{32}-{\dot{\mathrm{Ex}}}_{37}$	${\dot{\mathrm{Ex}}}_{F,\mathrm{IHE}}-{\dot{\mathrm{Ex}}}_{P,\mathrm{IHE}}$
Vapor condenser	${\dot{\mathrm{Ex}}}_{35}-{\dot{\mathrm{Ex}}}_{36}$	${\dot{\mathrm{Ex}}}_{39}-{\dot{\mathrm{Ex}}}_{38}$	${\dot{\mathrm{Ex}}}_{F,\mathrm{VC}}-{\dot{\mathrm{Ex}}}_{P,\mathrm{VC}}$
Pump 2	${\dot{\mathrm{Ex}}}_{46}$	${\dot{\mathrm{Ex}}}_{37}-{\dot{\mathrm{Ex}}}_{36}$	${\dot{\mathrm{Ex}}}_{F,\mathrm{pu}2}-{\dot{\mathrm{Ex}}}_{P,\mathrm{pu}2}$

**Table 5:** Cost balance and auxiliary equations for the system components.

Component	Cost Balance Equation	Auxiliary Equation
Air compressor	${\dot{C}}_{1}{+\dot{C}}_{40}{+\dot{Z}}_{\mathrm{AC}}\;{=\dot{C}}_{2}$	$c_{1}=0$
Air preheater	${\dot{C}}_{2}+{\dot{C}}_{8}+{\dot{Z}}_{\mathrm{AP}}={\dot{C}}_{3}+{\dot{C}}_{9}$	$\frac{{\dot{C}}_{8}}{{\dot{\mathrm{Ex}}}_{8}}=\frac{{\dot{C}}_{9}}{{\dot{\mathrm{Ex}}}_{9}}$
Gas turbine	${\dot{C}}_{3}{+\dot{Z}}_{\mathrm{GT}}\;{=\dot{C}}_{4}{+\dot{C}}_{40}{+\dot{C}}_{41}$	$\frac{{\dot{C}}_{3}}{{\dot{\mathrm{Ex}}}_{3}}=\frac{{\dot{C}}_{4}}{{\dot{\mathrm{Ex}}}_{4}},\frac{{\dot{C}}_{40}}{{\dot{\mathrm{Ex}}}_{40}}=\frac{{\dot{C}}_{41}}{{\dot{\mathrm{Ex}}}_{41}}$
Combustion chamber	${\dot{C}}_{4}{+\dot{C}}_{5}{+\dot{Z}}_{\mathrm{CC}}={\dot{C}}_{8}$	
Biomass gasifier	${\dot{C}}_{6}{+\dot{C}}_{7}{+\dot{Z}}_{\mathrm{Ga}}={\dot{C}}_{5}$	$c_{6}=0$
HRSG	${\dot{C}}_{9}{+\dot{C}}_{12}{+\dot{Z}}_{\mathrm{HRSG}}\;{=\dot{C}}_{10}{+\dot{C}}_{13}$	$\frac{{\dot{C}}_{9}}{{\dot{\mathrm{Ex}}}_{9}}=\frac{{\dot{C}}_{10}}{{\dot{\mathrm{Ex}}}_{10}}$
Steam turbine	${\dot{C}}_{13}{+\dot{Z}}_{\mathrm{ST}}\;{=\dot{C}}_{14}{+\dot{C}}_{42}$	$\frac{{\dot{C}}_{13}}{{\dot{\mathrm{Ex}}}_{13}}=\frac{{\dot{C}}_{14}}{{\dot{\mathrm{Ex}}}_{14}}$
Pump 1	${\dot{C}}_{15}{+\dot{C}}_{43}{+\dot{Z}}_{\mathrm{HP}}\;{=\dot{C}}_{12}$	$\frac{{\dot{C}}_{42}}{{\dot{\mathrm{Ex}}}_{42}}=\frac{{\dot{C}}_{43}}{{\dot{\mathrm{Ex}}}_{43}}$
Generator	${\dot{C}}_{14}{+\dot{C}}_{21}{+\dot{Z}}_{\mathrm{gen}}\;{=\dot{C}}_{15}{+\dot{C}}_{16}{+\dot{C}}_{22}$	$\frac{{\dot{C}}_{14}}{{\dot{\mathrm{Ex}}}_{14}}=\frac{{\dot{C}}_{15}}{{\dot{\mathrm{Ex}}}_{15}}$
		$\frac{{\dot{C}}_{16}-{\;\dot{C}}_{21}}{{\dot{\mathrm{Ex}}}_{16}-{\dot{\mathrm{Ex}}}_{21}}=\frac{{\;\dot{C}}_{22}-{\;\dot{C}}_{21}}{{\dot{\mathrm{Ex}}}_{22}-{\dot{\mathrm{Ex}}}_{21}}$
SHE	${\dot{C}}_{16}{+\dot{C}}_{20}{+\dot{Z}}_{\mathrm{SHE}}\;{=\dot{C}}_{17}{+\dot{C}}_{21}$	$\frac{{\;\dot{C}}_{16}}{{\dot{\mathrm{Ex}}}_{16}}=\frac{{\;\dot{C}}_{17}}{{\dot{\mathrm{Ex}}}_{17}}$
Absorber	${\;\dot{C}}_{18}{+\dot{C}}_{25}{+\dot{C}}_{30}{+\dot{Z}}_{\mathrm{abs}}={\dot{C}}_{19}{+\dot{C}}_{31}$	$\frac{{\dot{C}}_{19}}{{\dot{\mathrm{Ex}}}_{19}}=\frac{{\dot{C}}_{18}+{\dot{C}}_{25}}{{\dot{\mathrm{Ex}}}_{18}+{\dot{\mathrm{Ex}}}_{25}}{,\;c}_{30}=0$
Solution pump	${\dot{C}}_{19}{+\dot{C}}_{44}{+\dot{Z}}_{\mathrm{SP}\;}\;{=\dot{C}}_{20}$	$\frac{{\dot{C}}_{42}}{{\dot{\mathrm{Ex}}}_{42}}=\frac{{\dot{C}}_{44}}{{\dot{\mathrm{Ex}}}_{44}}$
Condenser	${\dot{C}}_{22}{+\dot{C}}_{26}{+\dot{Z}}_{\mathrm{con}}\;{=\dot{C}}_{23}{+\dot{C}}_{27}$	$\frac{{\dot{C}}_{22}}{{\dot{\mathrm{Ex}}}_{22}}=\frac{{\dot{C}}_{23}}{{\dot{\mathrm{Ex}}}_{23}}{,\;c}_{26}=0$
Evaporator	${\dot{C}}_{24}{+\dot{C}}_{28}{+\dot{Z}}_{\mathrm{eva}}\;{=\dot{C}}_{25}{+\dot{C}}_{29}$	$\frac{{\dot{C}}_{24}}{{\dot{\mathrm{Ex}}}_{24}}=\frac{{\dot{C}}_{25}}{{\dot{\mathrm{Ex}}}_{25}}{,\;c}_{28}=0$
Vapor generator	${\dot{C}}_{10}{+\dot{C}}_{32}{+\dot{Z}}_{\mathrm{VG}}={\dot{C}}_{11}{+\dot{C}}_{33}$	$\frac{{\dot{C}}_{10}}{{\dot{\mathrm{Ex}}}_{10}}=\frac{{\dot{C}}_{11}}{{\dot{\mathrm{Ex}}}_{11}}$
Vapor turbine	${\dot{C}}_{33}{+\dot{Z}}_{\mathrm{VT}}\;{=\dot{C}}_{34}{+\dot{C}}_{45}$	$\frac{{\dot{C}}_{33}}{{\dot{\mathrm{Ex}}}_{33}}=\frac{{\dot{C}}_{34}}{{\dot{\mathrm{Ex}}}_{34}}$
IHE	${\dot{C}}_{34}{+\dot{C}}_{37}{+\dot{Z}}_{\mathrm{IHE}}={\dot{C}}_{32}{+\dot{C}}_{35}$	$\frac{{\dot{C}}_{34}}{{\dot{\mathrm{Ex}}}_{34}}=\frac{{\dot{C}}_{35}}{{\dot{\mathrm{Ex}}}_{35}}$
Vapor condenser	${\dot{C}}_{35}{+\dot{C}}_{38}{+\dot{Z}}_{\mathrm{VC}}={\dot{\;C}}_{36}{+\dot{C}}_{39}$	$\frac{{\dot{C}}_{35}}{{\dot{\mathrm{Ex}}}_{35}}=\frac{{\dot{C}}_{36}}{{\dot{\mathrm{Ex}}}_{36}}{,\;c}_{38}=0$
Pump 2	${\dot{C}}_{36}{+\dot{C}}_{46}{+\dot{Z}}_{\mathrm{pu}2}\;{=\dot{C}}_{37}$	$\frac{{\dot{C}}_{45}}{{\dot{\mathrm{Ex}}}_{45}}=\frac{{\dot{C}}_{46}}{{\dot{\mathrm{Ex}}}_{46}}$

**Table 6:** Cost balance and auxiliary equations for the system components [42,43,44].

Component	Cost Balance Equation
Air compressor	$Z_{\mathrm{AC}}=71.1{\dot{m}}_{1}/(0.9-\eta_{\mathrm{is},\mathrm{AC}})\mathrm{In}(P_{2}/P_{1})$
Air preheater	$Z_{\mathrm{AP}}=4122{\left({\dot{m}}_{8}\left(h_{8}-h_{9}\right)/U_{\mathrm{AP}}/\Delta T_{\mathrm{lm},\mathrm{AP}}\right)}^{0.6}$
Gas turbine	$Z_{\mathrm{GT}}=479.34{\dot{m}}_{3}/\left(0.92-\eta_{\mathrm{is},\mathrm{GT}}\right)\mathrm{In}\left(P_{3}/P_{4}\right)\left[1+\exp(0.036T_{3}-54.4)\right]$
Combustion chamber	$Z_{\mathrm{CC}}=46.08{\dot{m}}_{4}/\left(0.995-P_{8}/P_{4}\right)\left[1+\exp(0.018T_{8}-26.4)\right]$
Biomass gasifier	$Z_{\mathrm{Ga}}=1600{\left({\dot{m}}_{\mathrm{dry}\;\mathrm{biomass}\;}[\mathrm{kg}/h]\right)}^{0.67}$
HRSG	$Z_{\mathrm{HRSG}}=6570\sum_{i}{\left({\dot{Q}}_{i}/\Delta T_{\mathrm{lm},i}\right)}^{0.8}{+21,276\dot{m}}_{12}+1184.4{{\dot{m}}_{9}}^{1.2}$
Steam turbine	$Z_{\mathrm{ST}}=6000{\left({\dot{W}}_{\mathrm{ST}}\right)}^{0.7}$
Pump 1	$Z_{\mathrm{pu}1}=3540{\left({\dot{W}}_{\mathrm{pu}1}\right)}^{0.71}$
Generator	$Z_{\mathrm{gen}}=17,500{\left(A_{\mathrm{gen}}/100\right)}^{0.6}$
SHE	$Z_{\mathrm{SHE}}=12,000{\left(A_{\mathrm{SHE}}/100\right)}^{0.6}$
Absorber	$Z_{\mathrm{abs}}=16,500{\left(A_{\mathrm{abs}}/100\right)}^{0.6}$
Condenser	$Z_{\mathrm{con}}=8000{\left(A_{\mathrm{con}}/100\right)}^{0.6}$
Evaporator	$Z_{\mathrm{eva}}=16,000{\left(A_{\mathrm{eva}}/100\right)}^{0.6}$
Solution pump	$Z_{\mathrm{SP}}=2100{\left({\dot{W}}_{\mathrm{SP}}/10\right)}^{0.26}{\left(\left(1-\eta_{\mathrm{is},\mathrm{SP}}\right)/\eta_{\mathrm{is},\mathrm{SP}}\right)}^{0.5}$
Vapor generator	$Z_{\mathrm{VG}\;}=130{\left(A_{\mathrm{VG}}/0.093\right)}^{0.78}$
Vapor turbine	$Z_{\mathrm{VT}\;}=6000{\left({\dot{W}}_{\mathrm{VT}}\right)}^{0.7}$
IHE	$Z_{\mathrm{IHE}}=1.3\left({190+310A}_{\mathrm{IHE}}\right)$
Vapor condenser	$Z_{\mathrm{VC}}\;{=1773\dot{m}}_{35}$
Pump 2	$Z_{\mathrm{pu}2}=3540{\left({\dot{W}}_{\mathrm{pu}2}\right)}^{0.71}$

**Table 7:** Results comparison between the present work and Ref. [48] for the EFGT cycle.

State	Substance	*P* (kPa)		*T* (K)		$\dot{m}$ (kg/s)	
		Ref. [48]	Present Work	Ref. [48]	Present Work	Ref. [48]	Present Work
1	Air	101.3	101.3	298.15	298.15	9.45	9.84
2	Air	911.7	911.7	589.9	583.84	9.45	9.84
3	Air	884.35	884.35	1400	1400	9.45	9.84
4	Air	103.83	103.88	877.6	886.18	9.45	9.84
5	Syngas	101.3	101.3	1073.15	1073.15	2.789	2.792
8	Comb. gas	102.82	102.84	1562	1578.6	12.24	12.63
9	Comb. gas	101.3	101.3	1000	1000	12.24	12.63

**Table 8:** Comparison of the component percentages of the syngas calculated by the present work with those reported in the literature (wood: CH_1.44_O_0.66_, MC = 20%, *T*_Ga_ = 1073.15 K).

Constituent	Roy et al. [19]	Cao et al. [49]	Present Work
H_2_ (%)	21.63	21.66	21.50
CO (%)	20.25	20.25	20.21
CH_4_ (%)	0.98	1.011	0.95
CO_2_ (%)	12.48	12.36	12.50
N_2_ (%)	44.94	44.72	44.84

**Table 9:** Results comparison between the present work and Ref. [50] for the ORC cycle with IHE using R601 as working fluid.

Parameter	*T*_eva_ (K)	*T*_con_ (K)	*P*_eva_ (kPa)	*P*_con_ (kPa)	$\dot{m}$ (kg/s)	*η*_th_ (%)
Ref. [50]	373.15	303.15	5.963	0.828	16.331	13.84
This work	373.15	303.15	5.927	0.820	16.382	13.84

**Table 10:** Results comparison between the present work and Ref. [32] for the single-effect LiBr-H_2_O ARC at same operating conditions (${\dot{Q}}_{\mathrm{eva}}$ = 3.51 kW, *T*_gen_ = 363.15 K, *T*_eva_ = 280.15 K, *T*_abs_ = 313.15 K, *T*_con_ = 313.15 K, $\varepsilon_{\mathrm{SHE}}$ = 0.8).

Parameter	Ref. [32]	This Work
Heat capacity of generator (kW)	4.5999	4.6000
Heat capacity of condenser (kW)	3.7432	3.7420
Heat capacity of absorber (kW)	4.368	4.368
Evaporator pressure (kPa)	1.0021	1.0021
Condenser pressure (kPa)	7.3844	7.3849
Weak solution concentration (%)	62.33	62.15
Strong solution concentration (%)	56.72	56.66
Refrigerant mass flow rate (kg/s)	0.0015	0.0015
Weak solution mass flow rate (kg/s)	0.0151	0.0154
Strong solution mass flow rate (kg/s)	0.0166	0.0169
Coefficient of performance	0.763	0.763

**Table 11:** Thermodynamic and exergoeconomic evaluation results of the base case.

Performance Parameters	Unit	Value
$\mathrm{SRC}\;\mathrm{turbine}\;\mathrm{work}\;({\dot{W}}_{\mathrm{ST}}$)	kW	4532.51
$\mathrm{SRC}\;\mathrm{pump}\;\mathrm{consumed}\;\mathrm{power}\;({\dot{W}}_{\mathrm{Pu}1}$)	kW	94.79
$\mathrm{ORC}\;\mathrm{turbine}\;\mathrm{work}\;({\dot{W}}_{\mathrm{VT}}$)	kW	527.95
$\mathrm{ORC}\;\mathrm{pump}\;\mathrm{consumed}\;\mathrm{power}\;({\dot{W}}_{\mathrm{VP}}$)	kW	15.45
$\mathrm{Net}\;\mathrm{power}\;\mathrm{output}\;({\dot{W}}_{\mathrm{net}}$)	kW	12,950.2
$\mathrm{Cooling}\;\mathrm{output}\;({\dot{Q}}_{\mathrm{eva}}$)	kW	7738.4
$\mathrm{Thermal}\;\mathrm{efficiency}\;(\eta_{\mathrm{th}}$)	%	70.67
$\mathrm{Exergy}\;\mathrm{efficiency}\;(\eta_{\mathrm{ex}}$)	%	39.13
$\mathrm{Unit}\;\mathrm{cost}\;\mathrm{of}\;\mathrm{the}\;\mathrm{GT}-\mathrm{produced}\;\mathrm{power}\;(c_{\mathrm{GT}}$)	USD/GJ	8.60
$\mathrm{Unit}\;\mathrm{cost}\;\mathrm{of}\;\mathrm{the}\;\mathrm{SRC}-\mathrm{produced}\;\mathrm{power}\;(c_{\mathrm{SRC}}$)	USD/GJ	15.60
$\mathrm{Unit}\;\mathrm{cost}\;\mathrm{of}\;\mathrm{the}\;\mathrm{ORC}-\mathrm{produced}\;\mathrm{power}\;(c_{\mathrm{ORC}}$)	USD/GJ	31.50
$\mathrm{Unit}\;\mathrm{cost}\;\mathrm{of}\;\mathrm{exergy}\;\mathrm{production}\;\mathrm{for}\;\mathrm{cooling}\;(c_{\mathrm{eva}}$)	USD/GJ	8.23
$\mathrm{LCOE}\;\mathrm{of}\;\mathrm{the}\;\mathrm{system}\;({\mathrm{LCOE}}_{\mathrm{sys}}$)	USD/GJ	11.67

**Table 12:** Selected decision variables of the proposed system and their limits.

Parameter	Unit	Range
*PR*_AC_	-	$6\;\leq {\;\mathrm{PR}}_{\mathrm{AC}}\;\leq \;16$
*T*_3_	K	$1100\;\leq {\;T}_{3}\;\leq \;1500$
CETD	K	200 ≤ CETD ≤ 300
Δ*T*_PP, HRSG_	K	$10\;\leq \;\Delta T_{\mathrm{PP},\;\mathrm{HRSG}}\;\leq \;50$
*P*_13_	kPa	$10,000\;\leq {\;P}_{13}\;\leq \;18,000$
*T*_15_	K	$358.15\;\leq {\;T}_{15}\;\leq \;368.15$
*P*_33_	kPa	$400\;\leq {\;P}_{33}\;\leq \;2000$

**Table 13:** The values of decision variables and objective functions at points A, B, and C.

Parameter	A	B	C
*PR*_AC_	11.03	7.86	10.62
*T*_3_ (K)	1479.2	1374.1	1450.2
CETD (K)	217.7	279.5	256.2
Δ*T*_PP, HRSG_ (K)	19.97	11.71	14.44
*P*_13_ (kPa)	16,509.9	10,257.5	16,642.3
*T*_15_ (K)	362.2	361.0	359.7
*P*_33_ (kPa)	1811.8	459.4	567.4
${\dot{W}}_{\mathrm{net}}\;$ (kW)	12,821.4	13,582.6	13,660.5
${\dot{Q}}_{\mathrm{eva}}\;$ (kW)	6863.4	9807.7	8771.8
*η*_ex_ (%)	39.40	35.66	38.15
*LCOE*_sys_ (USD/GJ)	11.74	10.59	11.01

**Table 14:** Comparison of thermodynamic efficiency and economic performance in current and previous investigations.

Parameter	Ref. [22]	This Work	Ref. [15]	This Work	Ref. [17]	This Work
*PR*_AC_	10		10		7	
GTIT (K)	1573.15		1300		1300	
Energy efficiency (%)	67.26%	71.72%	75.8%	68.93%	41.18%	64.62%
Exergy efficiency (%)	41.08%	41.31%	41.21%	36.58%	-	37.97%
Cost of products (USD/GJ)	17.17	19.32	10.2	11.74	-	-

## Data Availability

Data are contained within the article.

## Nomenclature

A	area (m^2^)	COP	coefficient of performance
c	cost per exergy unit (USD·GJ^−1^)	CRF	capital recovery factor
$\dot{C}$	cost rate (USD·h^−1^)	EFGT	externally fired gas turbine
*ex*	exergy per unit mass (kW·kg^−1^)	EV	expansion valve
$\dot{\mathrm{Ex}}$	exergy rate (kW)	eva	evaporator
*f*	exergoeconomic factor	GA	genetic algorithm
*h*	specific enthalpy (kJ·kg^−1^)	Ga	gasifier
*i_r_*	annual interest rate (%)	gen	generator
*K*	equilibrium constant	GT	gas turbine
$\dot{m}$	mass flow rate (kg·s^−1^)	GTIT	gas turbine inlet temperature
*n*	kilomoles of component (kmol)	HRSG	heat recovery steam generator
*N*	annual operating hours (h)	is	isentropic
*n_t_*	lifetime of the system	IHE	internal heat exchanger
*P*	pressure (kPa)	LCOE	levelized cost of exergy
$\dot{Q}$	heat transfer rate (kW)	LHV	lower heating value
*r*	relative cost difference	MW	molecular weight
*s*	specific entropy (kJ·kg^−1^·K^−1^)	ORC	organic Rankine cycle
*T*	temperature (K)	PR	pressure ratio
*U*	heat transfer coefficient (W·m^−2^·K^−1^)	pu	pump
$\dot{W}$	power (kW)	SHE	solution heat exchanger
$y_{D}$	exergy destruction ratio (%)	SP	solution pump
*Z*	investment cost (USD)	SRC	steam Rankine cycle
$\dot{Z}$	investment cost rate (USD/h)	ST	steam turbine
		STIP	steam turbine inlet pressure
Subscript and abbreviations		VC	vapor condenser
0	dead state	VG	vapor generator
1,2,…	state points	VT	vapor turbine
abs	absorber		
AC	air compressor	Greek Symbols	
AP	air preheater	Δ	difference
ARC	absorption refrigeration cycle	*η*	efficiency
CC	combustion chamber	*ε*	heat exchanger effectiveness
CETD	cold end temperature difference	*ϕ_r_*	maintenance factor
con	condenser	ψ	chemical exergy coefficient

## Appendix A

For the proposed system, input parameters for the subsystems under basic operating conditions are provided in Table A1. Table A2 details the thermodynamic and exergoeconomic properties of key state points, including temperature, pressure, mass flow rate, specific enthalpy, specific entropy, exergy, cost rate, and cost per exergy unit. Utilizing these parameters, the exergy and exergoeconomic indicators for the system components are calculated and presented in Table A3.

**Table A1:** Input parameters for the proposed system.

Parameter	Value	Unit
Reference temperature (*T*_0_)	298.15	K
Reference pressure (*P*_0_)	101.3	kPa
EFGT [12,51]		
$\mathrm{Air}\;\mathrm{compressor}\;\mathrm{isentropic}\;\mathrm{efficiency}\;(\eta_{\mathrm{is},\mathrm{AC}}$)	86	%
$\mathrm{Air}\;\mathrm{compressor}\;\mathrm{pressure}\;\mathrm{ratio}\;({\mathrm{PR}}_{\mathrm{AC}}$)	10	-
$\mathrm{Gas}\;\mathrm{turbine}\;\mathrm{isentropic}\;\mathrm{efficiency}\;(\eta_{\mathrm{is},\mathrm{GT}}$)	86	%
Gas turbine inlet temperature (*T*_3_)	1500	K
Cold end temperature difference (CETD)	245	K
Pressure drop of the cold side in the AP	5	%
Pressure drop of the hot side in the AP	3	%
Pressure drop of the flue gas in the CC	1	%
Pressure drop of the flue gas in the HRSG	5	%
Pressure drop of the flue gas in the VG	5	%
$\mathrm{Net}\;\mathrm{power}\;\mathrm{output}\;\mathrm{of}\;\mathrm{the}\;\mathrm{EFGT}\;({\dot{W}}_{\mathrm{EFGT}}$)	8000	kW
SRC [52,53]		
Turbine inlet pressure (*P*_13_)	15,000	kPa
Pinch point temperature difference of HRSG (Δ*T*_PP,HRSG_)	30	K
Condenser temperature (*T*_15_)	363.15	K
Steam quality at outlet of the ST	0.9	-
$\mathrm{Vapor}\;\mathrm{turbine}\;\mathrm{isentropic}\;\mathrm{efficiency}\;(\eta_{\mathrm{is},\mathrm{ST}}$)	85	%
$\mathrm{Pump}\;\mathrm{isentropic}\;\mathrm{efficiency}\;(\eta_{\mathrm{is},\mathrm{pu}1}$)	80	%
ARC [32]		
Generator temperature (*T*_16_)	358.15	K
Absorber temperature (*T*_19_)	308.15	K
Condenser temperature (*T*_23_)	308.15	K
Evaporator temperature (*T*_25_)	278.15	K
Effectiveness of solution heat exchanger	70	%
Cooling water inlet/outlet temperature in condenser (*T*_26_/*T*_27_)	298.15/303.15	K
Cooling water inlet/outlet temperature in evaporator (*T*_28_/*T*_29_)	285.15/280.15	K
Cooling water inlet/outlet temperature in absorber (*T*_30_/*T*_31_)	298.15/303.15	K
ORC [45]		
Turbine inlet pressure (*P*_33_)	1200	kPa
Condenser temperature (*T*_36_)	308.15	K
$\mathrm{Effectiveness}\;\mathrm{of}\;\mathrm{IHE}\;(\varepsilon_{\mathrm{SHE}}$)	90	%
$\mathrm{Turbine}\;\mathrm{isentropic}\;\mathrm{efficiency}\;(\eta_{\mathrm{is},\mathrm{VT}}$)	80	%
$\mathrm{Pump}\;\mathrm{isentropic}\;\mathrm{efficiency}\;(\eta_{\mathrm{is},\mathrm{pu}2}$)	80	%
Cooling water inlet/outlet temperature in condenser (*T*_38_/*T*_39_)	298.15/303.15	K

**Table A2:** Thermodynamic properties and costs of the streams for the proposed system.

State	Fluid	*T* (K)	*P* (kPa)	$\dot{m}$ (kg/s)	*h* (kJ/kg)	*s* (kJ·kg^−1^·K^−1^)	$\dot{\mathrm{Ex}}$ (kW)	$\dot{C}$ (USD/h)	*c* (USD/GJ)
1	Air	298.15	101.3	27.67	0	6.888	123.22	0	0
2	Air	605.05	1013.0	27.67	323.05	6.966	8421.49	325.11	10.72
3	Air	1500	962.35	27.67	1352.89	8.018	28,232.98	778.75	7.66
4	Air	978.07	116.88	27.67	740.68	8.125	10,415.24	287.28	7.66
5	Syngas	1073.15	101.3	5.12	−2710.35	10.10	28,904.59	240.45	2.31
6	Air	298.15	101.3	3.17	0	6.888	14.10	0	0
7	Biomass	298.15	101.3	1.95	−7104.72	-	34,044.75	210.78	1.72
8	Comb. gas	1557.97	115.72	32.78	201.88	8.840	31,417.46	594.83	5.26
9	Comb. gas	850.05	112.24	32.78	−667.18	8.108	10,084.42	190.93	5.26
10	Comb. gas	463.28	106.63	32.78	−1110.75	7.429	2178.85	41.25	5.26
11	Comb. gas	378.15	101.3	32.78	−1203.13	7.224	1158.43	21.93	5.26
12	Water	364.99	15,000	4.92	396.31	1.203	206.91	13.35	17.93
13	Water	787.84	15,000	4.92	3352.79	6.402	7125.33	202.91	7.91
14	Water	363.15	70.18	4.92	2431.28	6.850	1936.13	55.14	7.91
15	Water	363.15	70.18	4.92	377.04	1.193	127.63	3.63	7.91
16	LiBr/H_2_O	358.15	5.63	24.93	217.14	0.463	2087.64	73.44	9.77
17	LiBr/H_2_O	323.15	5.63	24.93	152.58	0.273	1888.97	66.45	9.77
18	LiBr/H_2_O	323.15	0.87	24.93	152.58	0.273	1888.97	66.45	9.77
19	LiBr/H_2_O	308.15	0.87	28.21	85.37	0.211	758.85	24.65	9.02
20	LiBr/H_2_O	308.15	5.63	28.21	85.37	0.211	758.85	24.66	9.03
21	LiBr/H_2_O	336.17	5.63	28.21	142.44	0.389	878.18	32.09	10.15
22	Water	358.15	5.63	3.27	2659.54	8.637	290.96	12.02	11.48
23	Water	308.15	5.63	3.27	146.63	0.505	1.93	0.08	11.48
24	Water	278.15	0.87	3.27	146.63	0.528	−20.26	−0.84	11.48
25	Water	278.15	0.87	3.27	2510.06	9.025	−576.68	−23.82	11.48
26	Water	298.15	101.3	393.63	104.92	0.367	0	0	0
27	Water	303.15	101.3	393.63	125.82	0.437	68.23	12.30	50.09
28	Water	285.15	101.3	368.84	50.51	0.181	450.89	0	0
29	Water	280.15	101.3	368.84	29.53	0.106	875.49	25.94	8.23
30	Water	298.15	101.3	459.98	104.92	0.367	0	0	0
31	Water	303.15	101.3	459.98	125.82	0.437	79.72	20.51	71.49
32	R601	337.80	1200	6.86	70.48	0.212	52.65	6.84	36.07
33	R601	407.57	1200	6.86	512.19	1.340	775.77	43.74	15.66
34	R601	351.24	97.70	6.86	435.19	1.396	134.14	7.56	15.66
35	R601	312.98	97.70	6.86	364.45	1.183	84.69	4.77	15.66
36	R601	308.15	97.70	6.86	−2.52	−0.008	2.60	0.15	15.66
37	R601	308.73	1200	6.86	−0.26	−0.007	15.06	3.11	57.37
38	Water	298.15	101.3	120.37	104.92	0.367	0	0	0
39	Water	303.15	101.3	120.37	125.82	0.437	20.86	5.22	69.56

**Table A3:** Exergy and exergoeconomic parameters of the system.

Component	${\dot{\mathrm{Ex}}}_{F,k}$ (kW)	${\dot{\mathrm{Ex}}}_{P,k}$ (kW)	${\dot{\mathrm{Ex}}}_{D,k}$ (kW)	$\eta_{\mathrm{ex},k}$ (%)	${\dot{Z}}_{k}$ (USD/h)	${\dot{C}}_{D,k}$ (USD/h)	*f_k_*	*r_k_*
Air compressor	8937.07	8298.28	638.80	92.85	48.27	19.79	70.93	0.265
Air preheater	21,333.04	19,811.49	1521.55	92.87	49.74	28.81	63.32	0.209
Gas turbine	17,817.75	16,937.07	880.67	95.06	33.18	24.29	57.73	0.123
Combustion chamber	39,319.83	31,417.46	7902.37	79.90	67.10	106.06	38.75	0.411
Biomass gasifier	34,058.85	28,904.59	5154.26	84.87	29.67	31.90	48.19	0.344
HRSG	7905.57	6918.42	987.15	87.51	39.88	18.69	68.09	0.447
Steam turbine	5189.20	4532.51	656.69	87.35	106.70	18.70	85.09	0.972
Pump 1	94.79	79.29	15.51	83.64	4.40	0.87	83.47	1.183
Generator	1808.51	1500.43	308.08	82.97	1.86	8.77	17.54	0.249
SHE	198.67	119.33	79.34	60.06	0.44	2.79	13.70	0.770
Absorber	553.45	79.72	473.72	14.41	2.54	15.39	14.17	6.923
Condenser	289.03	68.23	220.80	23.61	0.36	9.12	3.83	3.365
Evaporator	556.42	424.60	131.82	76.31	2.96	5.45	35.21	0.479
Vapor generator	1020.42	723.11	297.30	70.86	17.58	5.63	75.75	1.695
Vapor turbine	641.63	527.95	113.68	82.28	23.69	6.41	78.71	1.011
IHE	49.45	37.59	11.86	76.01	0.94	0.67	58.36	0.758
Vapor condenser	82.09	20.86	61.23	25.41	0.60	3.45	14.73	3.442
Pump 2	15.45	12.47	2.99	80.68	1.21	0.34	78.18	1.097

## Appendix B

The vapor generator stands as a critical component within the ORC, exerting a significant impact on the system efficiency. Within the vapor generator, the working fluid is heated by absorbing energy from the high-temperature exhaust gases. This process is characterized by a substantial disparity in the heat transfer coefficients between the exhaust (hot) side and the working fluid (cold) side. Given these conditions, a fin-and-tube heat exchanger (FTHE) is chosen for its superior heat transfer capabilities and enhanced stability during the recovery of waste heat from exhaust gases [46]. The geometric dimensions of the FTHE are detailed in Table A4.

**Table A4:** Geometric dimensions of the fin-and-tube heat exchanger.

Item	Value	Unit
Tube inner diameter, *d*_i_	20	mm
Tube outer diameter, *d*_o_	25	mm
Tube pitch, *S*_Tu_	60	mm
Fin height, *H*_F_	12.5	mm
Fin thichness, *δ*_F_	1	mm
Fin pitch, *Y*_F_	4	mm
Fouling factor [54,55]		
Exhaust gas, *r*_exh_	$1.7{\;\times \;10}^{-4}$	m^2^·K^−1^·W
Refrigerant (liquid), *r*_liq_	$1.761{\;\times \;10}^{-4}$	m^2^·K^−1^·W
Refrigerant (vapor), *r*_vap_	$3.522{\;\times \;10}^{-4}$	m^2^·K^−1^·W
Refrigerant (two-phase), *r*_tp_	$6.7{\;\times \;10}^{-4}$	m^2^·K^−1^·W
Tube row alignment	Staggered type	
Tube and fin material	Stainless steel 316L	

For the generation of saturated vapor, the vapor generator is mainly divided into preheating section and evaporating section The overall heat transfer coefficients for each section can be calculated by [56]:

(A1) $$\frac{1}{U_{\mathrm{FTHE}}}=\frac{\gamma }{\alpha_{i}}{+r}_{i}\gamma +\frac{\delta_{\mathrm{Tu}}\gamma }{\lambda_{\mathrm{Tu}}}+\frac{r_{o}}{\eta }+\frac{1}{\alpha_{o}\eta_{o}}$$

where *α*_i_ and *α*_o_ stand for heat transfer coefficient inside and outside the tube, respectively; *γ* represents the rib effect coefficient; *λ*_Tu_ denotes the thermal conductivity of the tube material; *r*_i_ and *r*_o_ refer to fouling resistances inside and outside the tube, respectively; *η*_o_ indicates the outside overall surface efficiency.

The Young correlation is employed to calculate the heat transfer coefficient of exhaust gas [57]:

(A2) $${\mathrm{Nu}}_{\mathrm{exh}}=0.1378(\frac{d_{b}G_{\max}}{\mu_{\mathrm{exh}}}{)}^{0.718}{{\mathrm{Pr}}_{\mathrm{exh}}}^{1/3}{\left(\frac{Y_{F}}{H_{F}}\right)}^{0.296}$$

For the single-phase flow in the tube side, the Gnielinski correlation is used [58]:

(A3) Nuwf =(f/8)(Rewf-1000)Prwf1+12.7f/8Prwf2/3-11+(diLTu)2/3ct

(A4) f=(1.82 lg Rewf-1.64)-2

For liquid state:

(A5) $$c_{t}=(\frac{{\mathrm{Pr}}_{\mathrm{wf}}}{{\mathrm{Pr}}_{\mathrm{wall}}}{)}^{0.01},\;\frac{{\mathrm{Pr}}_{\mathrm{wf}}}{{\mathrm{Pr}}_{\mathrm{wall}}}=0.05\sim 20$$

For vapor state:

(A6) $$c_{t}=(\frac{T_{\mathrm{wf}}}{T_{\mathrm{wall}}}{)}^{0.45},\;\frac{T_{\mathrm{wf}}}{T_{\mathrm{wall}}}=0.5\sim 1.5$$

The thermodynamic properties of ORC working fluid vary with the vapor quality for the two-phase flow on the tube side. In order to estimate the heat transfer area, the two-phase section is discretized and divided into 50 small parts so that the thermodynamic properties of the working fluid in each small part are considered to be constant. The convective heat transfer coefficient of the two-phase flow can be calculated by the Liu and Winterton correlation [59]:

(A7) $$h_{\mathrm{wf}}=\sqrt{{\left(F_{\mathrm{tp}}h_{\mathrm{liq}}\right)}^{2}+{\left(S_{\mathrm{tp}}h_{\mathrm{pool}}\right)}^{2}}$$

For the single:

(A8) $$F_{\mathrm{tp}}={\left[{1+x}_{\mathrm{wf}}{\mathrm{Pr}}_{\mathrm{liq}}\left(\frac{\rho_{\mathrm{liq}}}{\rho_{\mathrm{vap}}}-1\right)\right]}^{0.35}$$

(A9) Stp=(1+0.055Ftp0.1Reliq0.16)-1

(A10) hliq=0.023(λliq/do)Reliq0.8Prliq0.4

(A11) $$h_{\mathrm{pool}}{{=55\mathrm{Pr}}_{\mathrm{wf}}}^{0.12}{q_{\mathrm{wf}}}^{2/3}{\left(-\mathrm{lg}\;\mathrm{pr}\right)}^{-0.55}{{\mathrm{MW}}_{\mathrm{wf}}}^{-0.5}$$

As for the ARC, heat transfer coefficients of the main components are determined by adopting the values from the literature [35,60], as summarized in Table A5.

**Table A5:** Heat transfer coefficients of the components in the ARC.

Component	Heat Transfer Coefficient (W·m^−2^·K^−1^)
Generator	1500
Condenser	2500
Evaporator	1500
Absorber	700
SHE	1000
